# Comparative Study on Taste and Odor Flavor Compounds of Chrysanthemums from Five Different Varieties Using UPLC-Q-TOF-MS, GC-MS, E-Tongue, E-Nose, and Chemometrics

**DOI:** 10.3390/foods15183328

**Published:** 2026-09-19

**Authors:** Guizhou Hu, Xinqi Li, Geng Qin, Ailing Ben, Jin Qi

**Affiliations:** 1 School of Traditional Chinese Pharmacy, China Pharmaceutical University, Nanjing 211198, China; 2 School of Pharmacy, Bengbu Medical University, Bengbu 233030, China; 3 Jiangsu Provincial Key Laboratory for Plant Taxonomy, Resource Conservation and Utilization, Institute of Botany, Jiangsu Province and Chinese Academy of Sciences (Nanjing Botanical Garden Memorial Sun Yat-Sen), Nanjing 210014, China; 4 Nanjing Key Laboratory of Quality and Safety of Agricultural Products, College of Food Science, Nanjing XiaoZhuang University, Nanjing 211171, China

**Keywords:** Chrysanthemums, UPLC-Q-TOF-MS, GC-MS, E-tongue, E-nose, chemometrics

## Abstract

A practical strategy was established for distinguishing five varieties of Chrysanthemums by combining E-tongue, E-nose, GC-MS, and LC-Q-TOF-MS. A total of 19 low molecule fraction compounds and 26 volatile fraction compounds were identified as the common peaks. Chemometrics were used to screen the characteristic compounds of the five varieties of Chrysanthemums. E-tongue and E-nose were used to distinguish the taste and odor of the five varieties of Chrysanthemum, respectively. The Hangju variety had the richest taste flavor and the weakest aroma, while the Huaiju variety had the strongest odor of pine, woody, floral, and fruity aroma. The study found that 3, 5-dicaffeoylquinic acid, 1, 5-dicaffeoylquinic acid, luteolin-7-*β*-D-glucoside, and 4, 5-dicaffeoylquinic acid were the principal taste flavor components based on Gray Relational Analysis (GRA) and Partial Least Squares Regression (PLSR). *α*-pinene, camphene, *γ*-terpinene, camphor, etc. were identified as the principal volatile flavor components based on Spearman’s rank correlation. The combination of chemometrics and intelligent sensory technology presents a potential technique for analyzing the flavor of Chrysanthemums and other food products.

## 1. Introduction

Chrysanthemum, also known as *Chrysanthemum morifolium Ramat*, is a dried flower head that has been extensively utilized in China for over three millennia and was recognized as one of the ten most important traditional flowers [1]. Chrysanthemum contains rich chemical components, mainly flavonoids and volatile oils [2]. In China, Chrysanthemum has been extensively used as a traditional beverage and food in everyday life for centuries [3,4]. Specifically, one of the most popular teas among Chinese drinkers is Chrysanthemum tea, which is made from dried Chrysanthemums and soaked in hot water. According to diverse sources and various methods of processing, Chrysanthemum is divided into several commercial varieties, such as Boju (Bozhou, Anhui), Chuju (Chuzhou, Anhui), Gongju (Huangshan, Anhui), Hangju (Tongxiang, Zhejiang), and Huaiju (Jiaozuo, Henan) [2]. The differences in origins, environmental factors, harvesting, and processing methods can contribute to chemical composition variations among these five varieties of Chrysanthemums, which in turn leads to differences in aroma and flavor [5]. Chrysanthemum prices show a great difference among different geographical origins, and most consumers find it difficult to distinguish based on their appearance; as such, there can be market chaos and fraud [6]. Hence, it is imperative to employ a dependable analytical technique to evaluate the five varieties of Chrysanthemums.

To divide the quality of Chrysanthemums, Ouyang et al. assessed the discrepancies among various cultivars in the ingredient content through UPLC-MS/MS, GC-MS, and multivariate statistical analysis [7]. Nie et al. integrated UPLC-DAD/ESI-Q-TOF MS, GC–MS, and PCA analysis for quality evaluation and identification of cultivars of Chrysanthemi Flos (Juhua) [8]. Gao et al. used GC-MS, GC-O-MS, AEDA, and PLS-DA to characterize the aroma profiles and key aroma-active compounds of four Chinese chrysanthemum varieties [9]. Li et al. used sensory evaluation, HS-SPME-GC-MS, PCA, and OPLS-DA to characterize aroma differences and identify key aroma compounds among six chrysanthemum species [10]. These studies demonstrate that chromatographic and chemometric approaches are effective for characterizing chemical and aroma differences among Chrysanthemum varieties. However, most previous studies have focused primarily on chemical composition, aroma profiling, or varietal discrimination, whereas the comprehensive characterization of both odor and taste and their relationships with the underlying chemical composition remain comparatively limited.

The sensory characteristics of Chrysanthemum are determined by the combined effects of numerous chemical constituents. Although E-nose and E-tongue technologies have increasingly been applied in food analysis to provide objective and rapid measurements of odor and taste responses [11], most previous studies have primarily used these techniques for sensory profiling or sample discrimination [12,13]. Similarly, GC-MS and LC-Q-TOF-MS have been widely employed for the characterization of volatile and low-molecular-weight constituents, but their potential to explain the chemical basis of instrumental sensory responses has not been fully explored. Therefore, integrating sensory-oriented technologies with comprehensive chemical profiling and correlation-based chemometrics may provide a more informative approach for linking chemical composition with taste and odor characteristics. In this framework, E-nose and E-tongue capture the overall odor and taste response patterns, while GC-MS and LC-Q-TOF-MS provide complementary information on volatile and low-molecular-weight constituents, respectively [14]. Furthermore, GRA, PLSR, and Spearman correlation analysis can be used to bridge these analytical dimensions by identifying chemical constituents that are closely associated with specific instrumental sensory responses [15]. Such integration therefore shifts the focus from sample discrimination based on individual analytical platforms to the exploration of the chemical basis of sensory characteristics.

Hence, this research introduced an integrated approach that combining E-nose, E-tongue, and chemometrics to systematically characterize their chemical composition, odor, and taste profiles and to explore the relationships between sensory responses and chemical constituents. To start, the identification and screening of the low-molecular-weight and volatile compounds based on the five varieties of Chrysanthemums by using GC-MS, high-performance liquid chromatography–mass spectrometry (HPLC-Q-TOF-MS), and chemometrics were conducted. Then, the odor and taste profiles of five varieties of Chrysanthemums were achieved through E-nose and E-tongue. Gray Relational Analysis (GRA), Partial Least Squares Regression (PLSR), and Spearman’s rank correlation were employed to identify and evaluate the odor and volatile flavor compounds that contribute to the variations in flavor among five varieties of Chrysanthemums. The present study integrates sensory profiling with complementary chemical analyses and correlation-based chemometrics to establish links between taste, odor, and chemical composition. This integrated framework provides a more comprehensive understanding of the flavor characteristics of different Chrysanthemum varieties and offers potential chemical markers for quality differentiation and authenticity evaluation of Chrysanthemum products.

## 2. Materials and Methods

### 2.1. Reagents and Materials

The QL-3 Pure air pump and QL-300 Hydrogen generator were supplied by Shandong Saike-saisi Hydrogen Energy Co., Ltd. (Jinan, China). An AE-240 one in a million balance was used from Mettler Toledo Instruments Co., Ltd. (Zurich, Switzerland). The volatile oil tester was obtained from Chengdu Shuniu Co., Ltd. (Chengdu, China). Formic acid and methanol were obtained from Tedia (Fairfield, OH, USA). Ethyl acetate (analytical grade) was purchased from Yasheng Chemical Co., Ltd. (Wuxi, China). Chromatographic-grade acetonitrile also was obtained from Tedia (Fairfield, OH, USA). The purified water used in the experiment was provided by a millipore ultrapure water system (Millipore, Bedford, MA, USA). 1-Caffeoylquinic Acid (purity of 98%), Neochlorogenic Acid (purity of 98%), Chlorogenic acid (purity of 98%), Cryptochlorogenic Acid (purity of 98%), 1,3-Dicaffeoylquinic Acid (purity of 98%), 1,4-Dicaffeoylquinic Acid (purity of 98%), 3,4-Dicaffeoylquinic Acid (purity of 98%), 1,5-Dicaffeoylquinic Acid (purity of 98%), and 3,5-Dicaffeoylquinic Acid (purity of 98%) had been obtained from Nanjing Spring & Autumn Biological Engineering (Nanjing, China).

### 2.2. Tea Samples

#### 2.2.1. Collection

Ten independent batches of five commercial Chrysanthemum varieties (Boju, Chuju, Gongju, Hangju, and Huaiju), traditionally associated with five geographical origins, were obtained from the Kangmei herbal market in Bozhou (Anhui province, China), whose codes and origins are presented in Table S1 and Figure S1. All samples were stored under refrigerated conditions until analysis. All Chrysanthemum samples were authenticated by Professor Jin Qi, an expert in Traditional Chinese Medicine at China Pharmaceutical University, to ensure the accuracy of varietal identification before subsequent analyses.

#### 2.2.2. Extraction

The low molecule fractions and volatile fractions compounds of the Chrysanthemum samples were simultaneously extracted through high-temperature water steam distillation [16,17,18]. The dried Chrysanthemums samples were filtered through a sieve with a 40-mesh size after crushing, and then 50 g of powder was accurately weighed and transferred into a 1000 mL round-bottom flask. Distilled water (10-fold the weight of the sample) was added, and the mixture was soaked for 1 h. Then, 2 mL of ethyl acetate was added to the volatile oil collector, followed by heating and reflux extraction for 2 h.

After extraction, the volatile fractions were concentrated in the ethyl acetate layer [8]. All volatile samples were seal-stored at −20 °C. The aqueous layer was collected as the low-molecular-weight fraction. An aliquot of 1 mL of the aqueous extract was diluted twice with methanol and filtered through a 0.22 µm microporous membrane before LC–Q–TOF–MS analysis, and 100 μL of volatile oil extract was diluted 10 times with ethyl acetate and filtered through 0.22 μm organic microporous membrane for GC-MS analysis.

### 2.3. HPLC-Q-TOF-MS Analysis

#### 2.3.1. Chromatographic Conditions

An Agilent Infinity 1260 HPLC (Agilent, Santa Clara, CA, USA) was used to analyze the low molecule fraction compounds. A Merk Hibar-RP-18e column (250 mm × 4.6 mm, 5 µm, Tokyo, Japan) was used for chromatographic separation at a flow rate of 0.8 mL/min. The mobile phase consisted of two solvents: solvent A, which was composed of 0.1% formic acid in water, and solvent B, which was acetonitrile. The volume of the injected sample was 10 µL, and the column temperature was consistently maintained at 30 °C. The gradient was programmed like this: 8–17% B in 0~15 min, 17–18% B in 15~30 min, 18–22% B in 30~50 min, 22%~40% B in 50~65 min, and 40%~50% B in 65~80 min. The wavelength for detection was adjusted to 350 nm.

#### 2.3.2. Conditions of Mass Spectrometry

An Agilent 6530 HPLC-Q-TOF mass spectrometer equipped with an electrospray ionization (ESI) interface was used in MS analysis with the same conditions as for HPLC analysis. The mass spectrometer was utilized with the following parameters: the capillary temperature was set to 350 °C, the drying gas (N_2_) flow rate was 10.0 L/min, the atomization pressure was 40 psi, the temperature was 320 °C, the Source CID was 12 V, and the capillary voltage was 3500 V. The MS acquisition parameters were identical for both positive and negative ion modes, with a scanning range of *m*/*z* 100–1500. All acquired data were processed using Mass Hunter Workstation Qualitative Analysis Software (version B.07.00; Agilent Technologies, Santa Clara, CA, USA), including peak extraction, alignment, and normalization. Finally, retention time, accurate mass-to-charge ratio, and peak area were outputted. The compound’s peaks were identified based on their molecular weights, MS/MS spectra, retention time, and elemental compositions using the structural characterization database. The database consists of SciFinder, ChemSpider (http://www.chemspider.com) (accessed on 16 September 2026), MassBank (http://www.massbank.jp/) (accessed on 16 September 2026), and PubChem (PubChem) (accessed on 16 September 2026).

### 2.4. GC–MS Analysis

#### 2.4.1. Conditions of GC

The volatile compounds were analyzed using a Bruker GC-456 gas chromatograph (Bruker, Karlsruhe, Germany) and a BR-1701 hairlike column (30 m × 0.25 mm, 0.25 µm). The tail-blowing process involved the use of N_2_ gas with a purity level exceeding 99.999% flowing at a rate of 30 mL/min. Hydrogen (H_2_) and air were supplied to the FID at flow rates of 30 and 300 mL/min, respectively. Helium gas was employed as the carrier gas flowing rate of 1 mL/min, and the injector temperature was maintained at 250 °C. Approximately 1 µL aliquots were injected with a split ratio of 1:10. The FID detector was utilized, with the detector temperature set at 280 °C. Initially, the temperature was set at 50 °C for 5 min. The temperature was programmed as follows: increased to 80 °C with a rate of 5 °C/min for 6 min, increased to 100 °C with a rate of 2 °C/min for 10 min, raised to 110 °C with a rate of 2 °C/min for 5 min, raised to 120 °C with a rate of 2.5 °C/min for 4 min, increased to 125 °C with a rate of 1.5 °C/min for 8 min, increased to 130 °C with a rate of 1 °C/min for 5 min, increased to 150 °C with a rate of 2.5 °C/min for 8 min, and finally increased to 250 °C with a rate of 5 °C/min for 20 min. GC-FID was used to obtain the chromatographic fingerprints of the volatile fractions of the five Chrysanthemum varieties, and the similarities among the fingerprints were evaluated.

#### 2.4.2. Conditions of Mass Spectrometry

An Agilent 8890-7000D GC-QQQ (Agilent, CA, USA) mass spectrometer and capillary column (BR-1701, 30 m × 0.25 mm, 0.25 µm) were used for the qualitative characterization of volatile fractions. The temperature program was the same as that described in Section 2.4.1. The ionization voltage on the mass spectrometer was set at 70 eV with a solvent delay time of 5 min. The electron ionization (EI) ion source was maintained at a temperature of 280 °C with a scanning range of *m*/*z* 40 to 500. The volatile compounds were identified by comparing them with the mass spectral library of the National Institute of Standards and Technology (NIST17) MS database. The mass spectral data were analyzed using the NIST-matched automated mass spectral inverse plethysmography identification system (AMDIS). GC-MS data were subsequently used for the qualitative identification of the volatile compounds based on their mass spectral characteristics and database matching.

### 2.5. Characteristic Compound Analysis

#### 2.5.1. Similarity Evaluation and Hierarchical Cluster Analysis of the Fingerprints

The LC-HRMS and GC-MS data, including peak areas and retention times, were exported in AIA (*.CDF) format. The HPLC and GC-FID data were analyzed using the Similarity Evaluation System for Chromatographic Fingerprint of Traditional Chinese Medicine (2012 Edition and 2004 Edition), respectively, for chromatographic alignment and similarity evaluation. Then, the fingerprints of the low molecule fractions and volatile fractions of 50 batches of Chrysanthemums were established, and the common peaks were analyzed using hierarchical cluster analysis (HCA) to classify the new matrix data and determine the similarities and differences. HCA is an unsupervised pattern recognition method that visually represents the similarities and differences among the test samples [19].

#### 2.5.2. Screening of Characteristic Compounds

The normalized data were subjected to unsupervised principal component analysis (PCA) and supervised orthogonal partial least squares discriminate analysis (OPLS-DA) using SIMCA-P14.0 software (Umetrics, Sweden). PCA was initially performed to visualize the overall distribution of the samples and assess the intrinsic clustering patterns among the five Chrysanthemum varieties without incorporating predefined class information. OPLS-DA was subsequently performed to enhance discrimination among the five predefined varieties and to identify variables contributing to the observed inter-variety differences.

The OPLS-DA model was evaluated using a 7-fold cross-validation procedure. Briefly, the dataset was divided into seven subsets, with six subsets used for model construction and the remaining subset used for validation in each iteration. This procedure was repeated until each subset had been used once for validation [20,21]. The goodness of fit and predictive ability of the model were evaluated using R^2^ and Q^2^, respectively [13]. No independent external validation dataset was used in this study; therefore, 7-fold cross-validation represented an internal validation procedure. Variable Importance in the Projection (VIP > 1) was employed to identify significant differential variables by assessing their respective contributions to the observed differences.

### 2.6. E-Tongue and E-Nose Analysis

The taste profiles of Chrysanthemum samples were acquired using the E-tongue α-ASTREE (Alpha MOS Company, Toulouse, France), which consisted of seven electrodes (AHS, CTS, NMS, ANS, SCS, PKS, CPS). Additionally, an Ag/AgCl reference electrode was employed in the experimental setup. The output value was considered by the average of the measurements during the last 20 s. The experiment was conducted six times, and the data collected from the last three measurements were used for subsequent data analysis. The data obtained from the E-tongue were subjected to PCA and discriminant factor analysis (DFA) using Alphasoft v14.20 software. First, 2 g of Chrysanthemum samples (not powder) was accurately measured in a beaker. Subsequently, 200 mL of double-distilled water that had been boiled was added to the beaker. After being immersed for 10 min, the Chrysanthemum sample was subjected to extraction using a 0.22 µm filter membrane. The filtrate was subsequently cooled to room temperature, and an aliquot (100 mL) was collected for E-tongue analysis.

The analysis of volatile information was conducted utilizing a PEN 3.5 E-nose manufactured by Airsense Analytics, GmBH, Germany. The E-nose model was equipped with ten sensors, and the sensor’s description is given in Table S2. First, 0.25 g Chrysanthemum powder was placed in a 50 mL beaker. The sample was sealed with tin foil for 30 min to facilitate the generation of gaseous compounds in the headspace at room temperature. The gaseous compounds were drawn into a sensor chamber at a consistent flow rate of 600 mL/min. The collection phase lasted for 200 s and cleaned before subsequent measurement. The data obtained from this process were subjected to PCA, linear discriminant analysis (LDA), and loading analysis (LOA) using Win Muster software (Version 1.6.2) on the G/G0 data.

### 2.7. Flavor Compounds

#### 2.7.1. Screening Crucial Flavor Compounds

Taste flavor compounds of Chrysanthemum were obtained through GRA and PLSR. The common peak areas of the low molecule fractions of Chrysanthemum were independent variables, and the E-tongue sensor response values of each sample were dependent variables. GRA is a statistical method to study the correlation between factors, emphasizing the order of correlation. The higher the gray grade (GRD), the closer the relationship. The PLSR analysis is a multiple regression model, combining multiple data fusion and principal component analysis.

Volatile flavor compounds of Chrysanthemum were screened based on these correlation coefficients, which meant the contribution of volatile fractions to the odor of Chrysanthemum teas with Nonparametric Spearman’s correlation using SPSS 22.0 software (Chicago, IL, USA). The independent variables were the common peak areas of the volatile components of Chrysanthemum, and the dependent variables were the response values of the E-nose sensor for each sample.

#### 2.7.2. Network Visualization

Cytoscape_v3.9.0 was employed to construct a network that represents the connections between E-nose/E-tongue sensors and volatile/taste flavor compounds. The utilization of network visualization analysis serves as an effective approach for investigating potential associations between sensor properties and flavor compounds.

## 3. Results and Discussion

### 3.1. Screening the Low Molecule Fraction and the Volatile Fraction Compounds of Five Varieties of Chrysanthemums

#### 3.1.1. Qualitative Analysis

A total of 42 low-molecular-weight compounds were identified from five varieties of Chrysanthemums (Figure S2 and Table S3), including 30 flavonoids, 11 phenylpropanoids, and one amino acid. Flavonoids and caffeoylquinic acid derivatives represent the major phenolic constituents in Chrysanthemum, which have been widely reported by LC-MS-based chemical profiling studies [22]. The difference was mainly in flavonoids, among which Boju, Chuju, Gongju, Hangju, and Huaijiu contained 26, 24, 20, 21, and 24 flavonoids, respectively. Except for the Gongju variety, which lacked one phenylpropanoid component, peak 15 (1,4-Dicaffeoylquinic Acid), the rest contained eleven phenylpropanoids and one amino acid. Ouyang et al. have also demonstrated that chemical profiles, especially flavonoid and phenolic acid compositions, vary among different Chrysanthemum varieties [7].

As displayed in Figures S3 and S4A and Table S4, 186 peaks were found and 162 compounds of the volatile fraction compounds were identified, which containing 53 olefins, 51 alcohols, 18 ketones, 11 esters, 10 aldehydes, six ether, four carboxylic acids, three aliphatic, three aromatic, two terpenoids, and one phenolic. Olefins (33%), alcohols (31%), and ketones (11%) were the top three major components of Chrysanthemums, and the three made up 75%. As shown in Figure S4B, Boju, Chuju, Gongju, Hangju, and Huaijiu varieties contained 60, 73, 58, 71, and 56 components, indicating that the constituent species of volatile fraction compounds from five varieties of Chrysanthemums do not differ from each other. The relative content of volatile compounds was calculated using the peak area normalization method.

#### 3.1.2. Fingerprint and HCA of the Low Molecule Fractions and the Volatile Fractions

**Figure 1:**
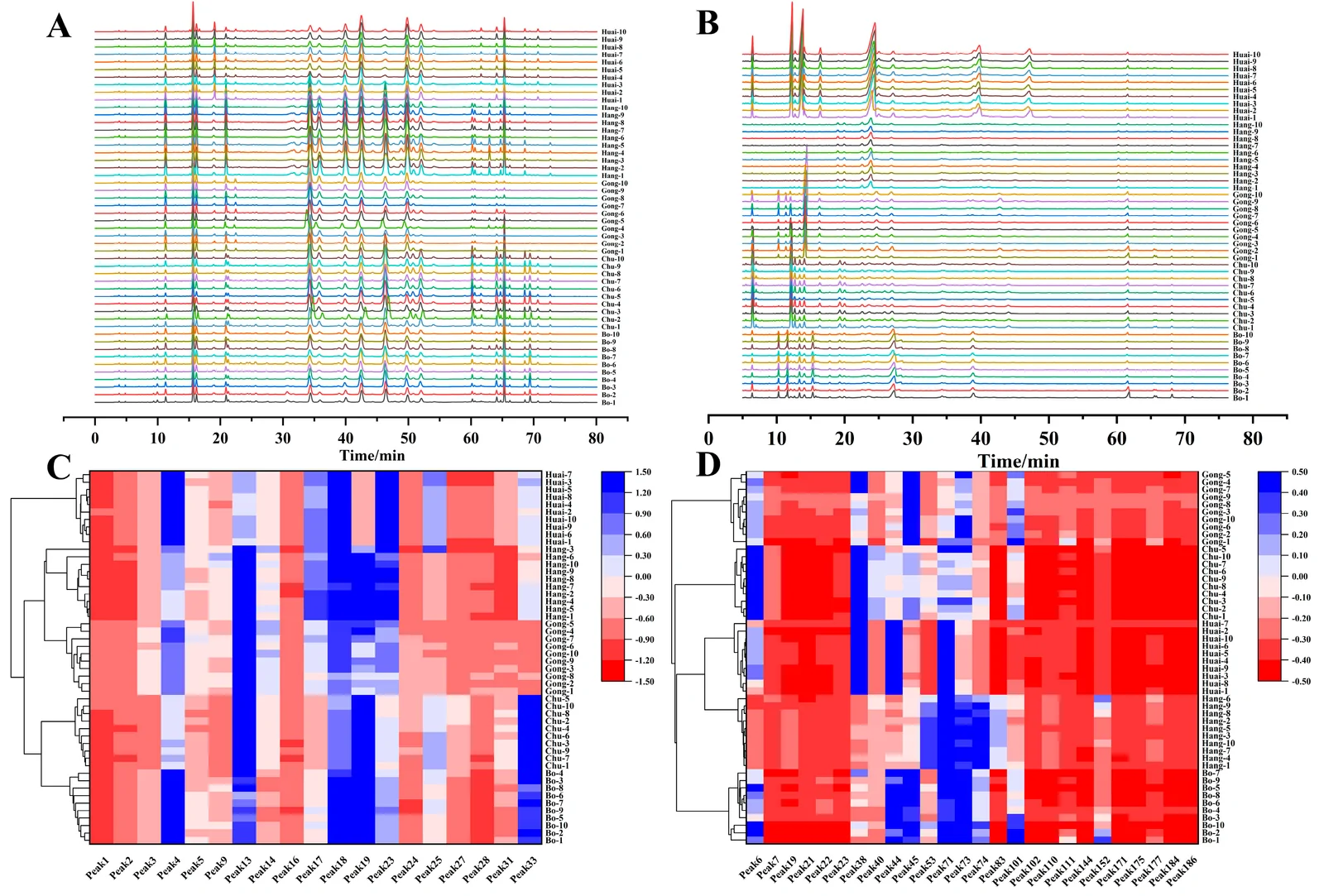
The HPLC chromatographic fingerprint analysis and clustering analysis of five Chrysanthemums varieties (*n* = 50). (**A**): HPLC fingerprint profiles of low molecule fractions from five Chrysanthemum varieties. (**B**): GC fingerprint profiles of volatile fractions from five Chrysanthemum varieties. (**C**,**D**): Heatmap visualization and HCA of low molecule fractions (**C**) and volatile fractions (**D**) based on the chromatographic peak intensities of the five Chrysanthemum varieties. The colors represent the relative intensity of chromatographic peaks, with red and blue indicating higher and lower abundance, respectively. Bo, Boju variety; Chu, Chuju variety; Gong, Gongju variety; Hang, Hangju variety; Huai, Huaiju variety.

For the low molecule fraction compounds, Figure 1A shows the LC fingerprints of 50 samples, whose similarity was greater than 0.85 (Table S5), indicating a high degree of compositional similarity among the five varieties of Chrysanthemum. Nineteen significant peaks (Figure 1C) exhibited distinct separations that were identified as the shared characteristic peaks. The area of non-shared peaks in each sample accounted for less than 10% of the total peak area, thereby satisfying the criteria for establishing a reliable fingerprint [23]. Five varieties of Chrysanthemums were approximately classified into two distinct categories according to the HCA (Figure 1C). The initial classification pertained to the Huaiju variety, and the other four classifications fell under the second category. Within the second group, there were subdivisions of Hangju and Gongju, Chuju, and Boju varieties. This showed that the internal quality of the five Chrysanthemum samples exhibited relative stability, with noticeable differences between varieties. The clustering trend was consistent with the similarity results, and the results obtained were mutually verified.

For the volatile fraction compounds, Figure 1B showed the GC fingerprints of 50 samples, whose similarity was between 0.382 and 0.928 (Table S6), suggesting that there were notable variations in the specific constituents among the five Chrysanthemum varieties despite their fundamental similarity in compound types. Twenty-six prominent peaks (Figure 1D) exhibited distinct separations that were identified as the shared characteristic peaks. HCA results showed that Hangju and Huaiju varieties were first clustered into one category as the clustering threshold increased, and then Boju and Chuju varieties were added in turn. Finally, they were divided into two major categories, with the Gongju variety in a separate category. These results were consistent with the results of the similarity analysis, demonstrating a correlation between chemical composition and chrysanthemum diversity.

#### 3.1.3. Screening the Low Molecule Fraction and the Volatile Fraction Compounds

##### The Low Molecule Fraction Compounds

**Figure 2:**
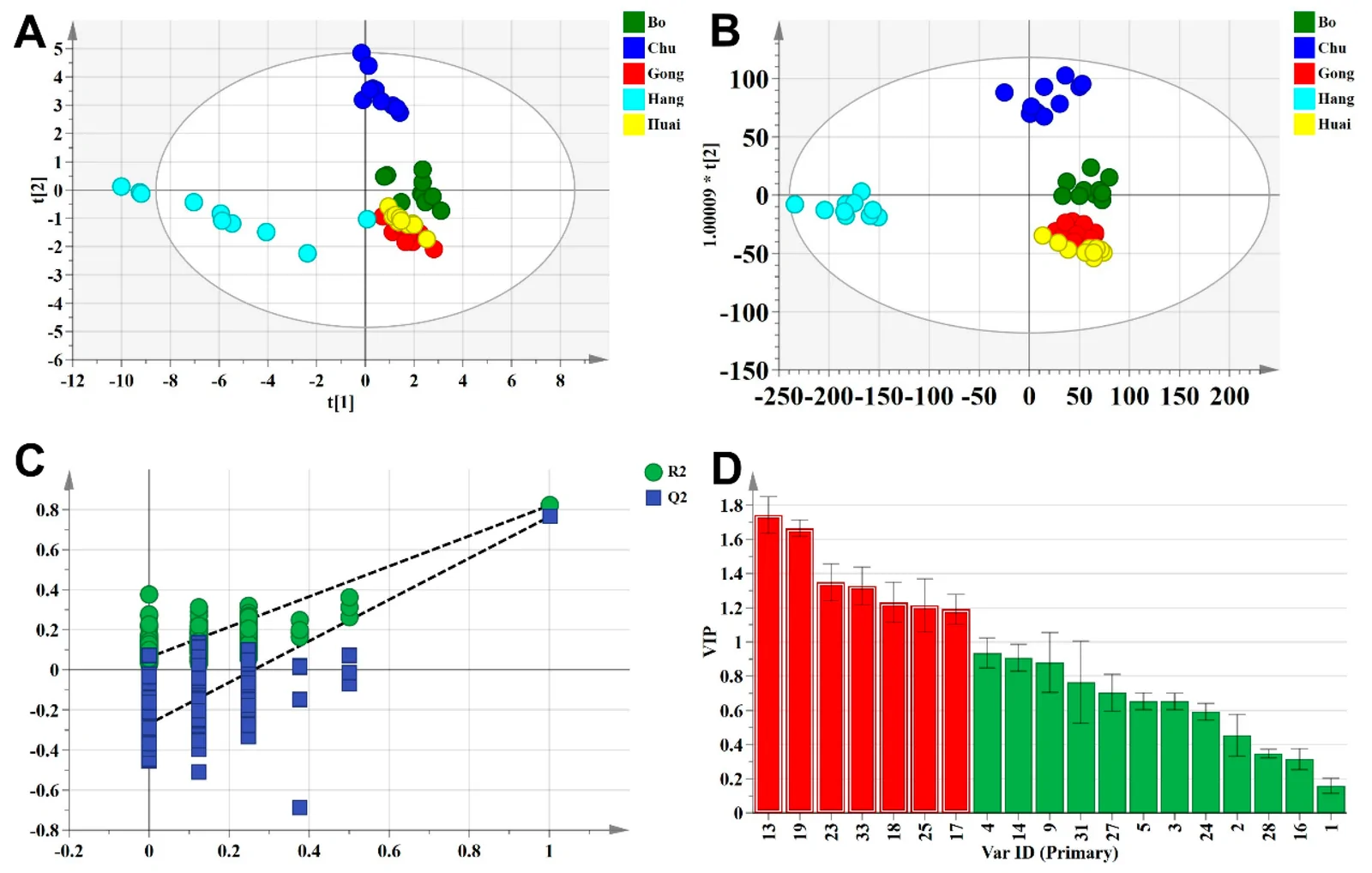
Multivariate statistical analysis of 19 shared characteristic peaks from low molecule fraction compounds among five Chrysanthemum varieties. (**A**): PCA score plot showing the overall distribution of the samples. (**B**): OPLS-DA score plot showing the discrimination among the five Chrysanthemum varieties. (**C**): Permutation test for validation of the OPLS-DA model. (**D**): VIP plot showing the contribution of individual variables to the discrimination among Chrysanthemum varieties. Variables with VIP > 1 were considered important compounds. Bo, Boju variety; Chu, Chuju variety; Gong, Gongju variety; Hang, Hangju variety; Huai, Huaiju variety.

For the low molecule fraction compounds, the PCA of five varieties of Chrysanthemums is shown in Figure 2A. The five varieties of Chrysanthemums exhibited distinct distribution patterns in the PCA score plot. Hangju and Chuju varieties were completely separated from other three Chrysanthemums varieties, whereas Boju, Huaiju, and Gongju varieties partially intersected in the distribution regions. These results suggest differences in the overall chemical profiles among the five Chrysanthemum varieties. Further supervised OPLS-DA was performed (Figure 2B), whose R^2^X = 0.986, R^2^Y = 0.901, and Q^2^ = 0.856, and all samples fell within 95% confidence intervals; the five varieties of Chrysanthemum were more completely separated in their spatial distributions. The expiation and validity of the established model were further demonstrated through the implementation of a seven-cross validation analysis, resulting in R^2^X = 0.978, R^2^Y = 0.923, and Q^2^ = 0.867 (Figure 2C). Finally, seven key compounds were screened based on their VIP (Figure 2D), which were luteolin-7-*β*-D-glucoside (P13), apigenin-7-*β*-D-glucoside (P19), 4,5-dicaffeoylquinic acid (P23), acacetin-7-*β*-D-glucoside (P33), 3,5-dicaffeoylquinic acid (P18), diosmetin-7-*β*-D-glucoside (P25), and 1,5-dicaffeoylquinic acid (P17). Xie et al. have demonstrated that flavonoid glycosides and caffeoylquinic acid derivatives are major characteristic compounds contributing to the discrimination of different Chrysanthemum varieties, which is consistent with the present study [24].

##### The Volatile Fraction Compounds

**Figure 3:**
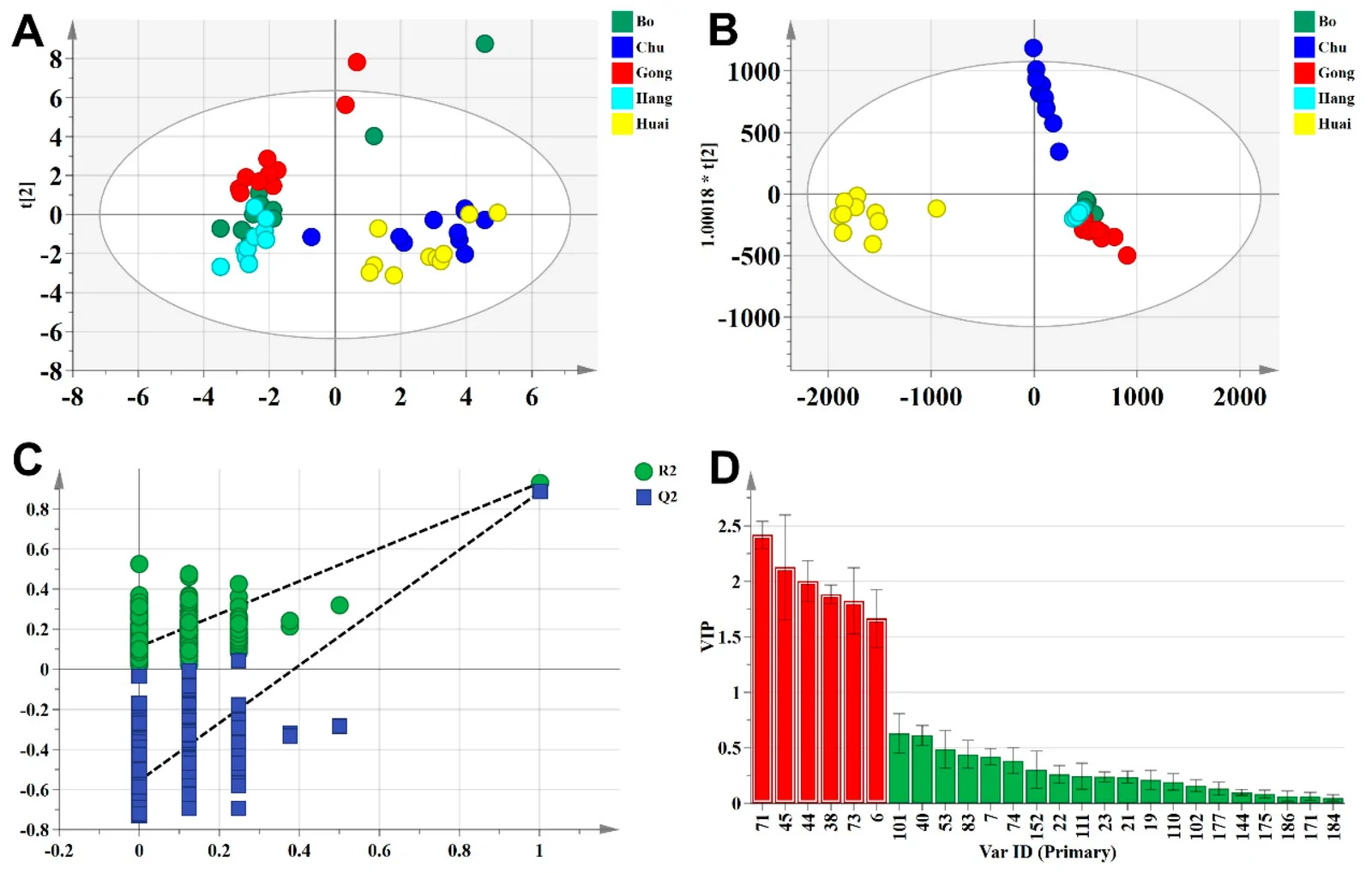
Multivariate statistical analysis of 26 shared characteristic peaks from volatile fraction compounds among five Chrysanthemum varieties. (**A**): PCA score plot showing the overall distribution of the samples. (**B**): OPLS-DA score plot showing the discrimination among the five Chrysanthemum varieties. (**C**): Permutation test for validation of the OPLS-DA model. (**D**): VIP plot showing the contribution of individual variables to the discrimination among Chrysanthemum varieties. Variables with VIP > 1 were considered important compounds. Bo, Boju variety; Chu, Chuju variety; Gong, Gongju variety; Hang, Hangju variety; Huai, Huaiju variety.

For the volatile components, the PCA of five varieties of Chrysanthemums is depicted in Figure 3A, which shows that the distributions of the five varieties of Chrysanthemums in the space of principal components were still partially crossed. Further supervised OPLS-DA was performed (Figure 3B), whose R^2^X = 0.977, R^2^Y = 0.92, and Q^2^ = 0.88, showing that Chuju and Huaiju varieties were completely separated from other Chrysanthemum varieties, indicating distinct overall chemical profiles among these varieties. The remaining three Chrysanthemums still had partial overlap, but all of them were further separated compared with the results of the PCA. Furthermore, the reliability of the established model was demonstrated through the implementation of a seven-cross validation analysis, resulting in R^2^X = 0.933, R^2^Y = 0.946, and Q^2^ = 0.92 (Figure 3C). Six key compounds were screened based on their VIP values (Figure 3D). These compounds include *β*-caryophyllene (P71), *α*-terpineol (P45), terpinen-4-ol (P44), camphor (P38), humulene (P73), and *α*-pinene (P6). Yang et al. have demonstrated that monoterpenes and sesquiterpenes are important volatile constituents contributing to the characteristic aroma profiles of different Chrysanthemum varieties [25].

### 3.2. Comparison of E-Tongue and E-Nose of Five Varieties of Chrysanthemums

#### 3.2.1. E-Tongue

**Figure 4:**
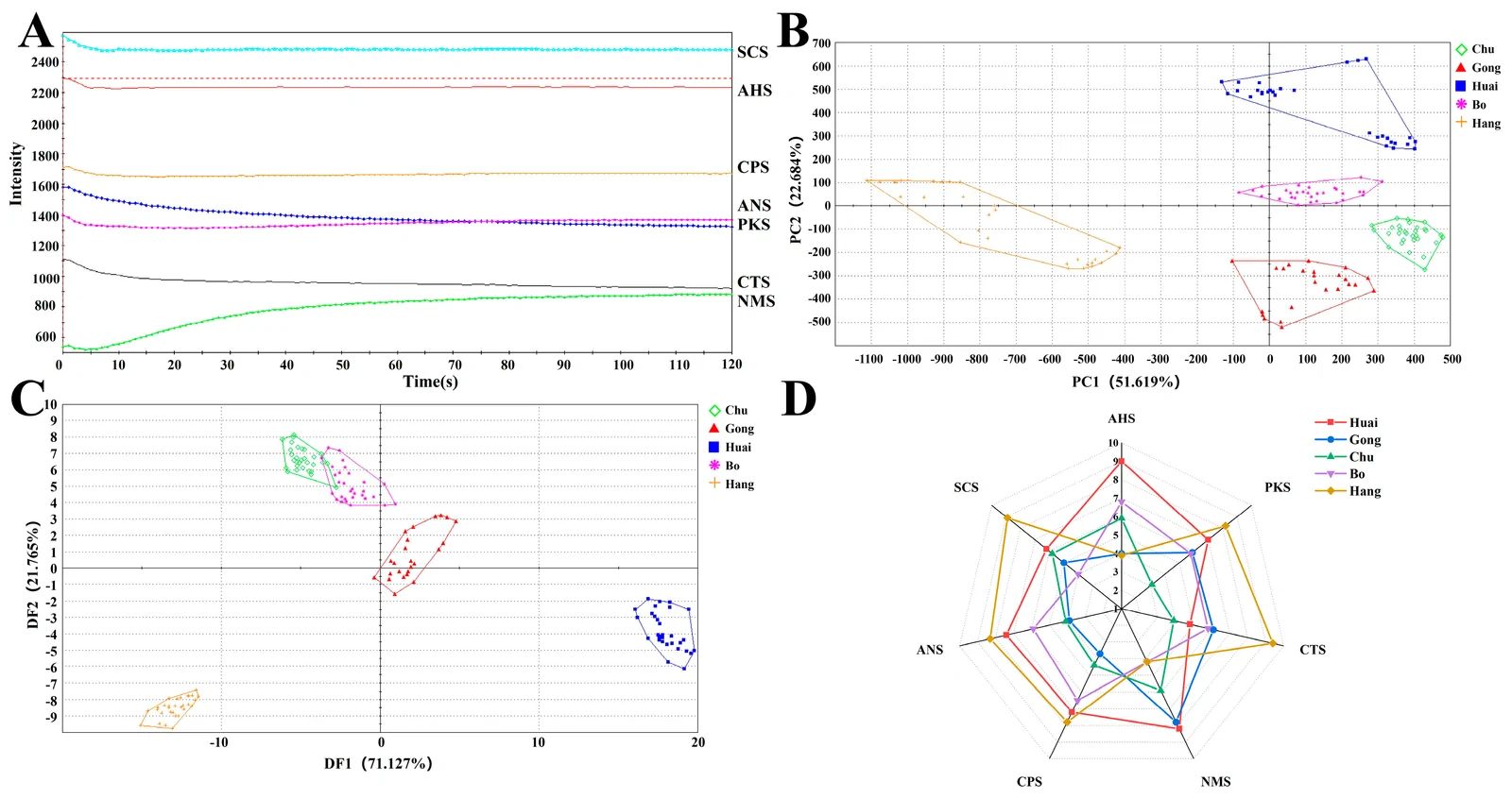
Taste response profiles of five Chrysanthemum varieties analyzed using an electronic tongue. (**A**) Response curves of electronic tongue sensors to Chrysanthemum sample solutions, including SCS (bitterness), ANS (sweet), CPS (the integrated sensor), PKS (the integrated sensor), CTS (saltiness), AHS (sourness), and NMS (umami). (**B**) PCA score plot based on electronic tongue response signals. (**C**) DFA score plot showing the discrimination among five Chrysanthemum varieties. (**D**) Radar response maps illustrating the overall taste characteristics of different Chrysanthemum varieties. Bo, Boju variety; Chu, Chuju variety; Gong, Gongju variety; Hang, Hangju variety; Huai, Huaiju variety.

The response curves of the sample solution on each sensor are shown in Figure 4A. The *X*-axis is measurement time, and the *Y*-axis represents the collective signal intensity value of the voltage difference between the sensor and the reference electrode. The response signal gradually stabilized in 100–120 s. The average value of the last 20 s of measurement was chosen as the raw data for subsequent data processing.

PCA and Discriminant Function Analysis (DFA) of the flavor response signals of five varieties of Chrysanthemums are shown in Figure 4B,C, respectively. The first principal component (PC1) accounted for 56.619% of the total variance, while the second principal component (PC2) explained 22.684% of the variance. The first discriminant factor (DF1) and the second discriminant factor (DF2) were identified as the primary influencing factors, and their cumulative contribution reached 92.892%. This suggested that the obtained results were highly reliable and that E-tongue was capable of distinguishing between the tastes of five different Chrysanthemums. The dispersion between the samples of different varieties was large, which indicated that E-tongue showed good differentiation between the different varieties of Chrysanthemums and that each Chrysanthemum had a unique flavor.

The flavor characteristics of five Chrysanthemum varieties varied across the five regions, as indicated by the radar response maps (Figure 4D). Hangju and Huaiju varieties were stronger than the other three Chrysanthemums in the sensors SCS (bitterness), ANS (sweet), CPS (the integrated sensor), and PKS (the integrated sensor). In AHS (sourness) and NMS (umami), Huaiju was also ranked first in strength. In the sensor CTS (saltiness), Hangju had a greater performance. Boju was more sour, salty, and sweet, Chuju was more sour, fresh, and bitter, and Gongju was more salty and fresh. Therefore, Huaiju had obvious sourness and freshness, and Hangju was the strongest among the five varieties of Chrysanthemums in terms of sweetness, bitterness, saltiness, and composite flavor. As the most common variety on the market [2,26], the Hangju variety is known as the best-tasting tea by consumers in China, which may be related to its rich taste.

The detection object of E-tongue was Chrysanthemum tea soup, which mainly dissolved soluble non-volatile components during the hot water soaking process. In Section 3.1.3, the findings indicate that there were discernible variations in the non-volatile constituents across various sources of Chrysanthemum. So, it was speculated that non-volatile components may be the material basis for the taste differences of the five kinds of Chrysanthemum. In conclusion, this suggested that the E-tongue was a promising tool for identifying Chrysanthemums of five different origins.

#### 3.2.2. E-Nose

The response curves of ten sensors from the E-nose are depicted in Figure S5. In this representation, the *X*-axis denotes the time of detection, and *Y*-axis signifies the value of the detection response signal (G/G0). The response signal value between 190 and 200 s was chosen as the primary data for further analysis [27].

**Figure 5:**
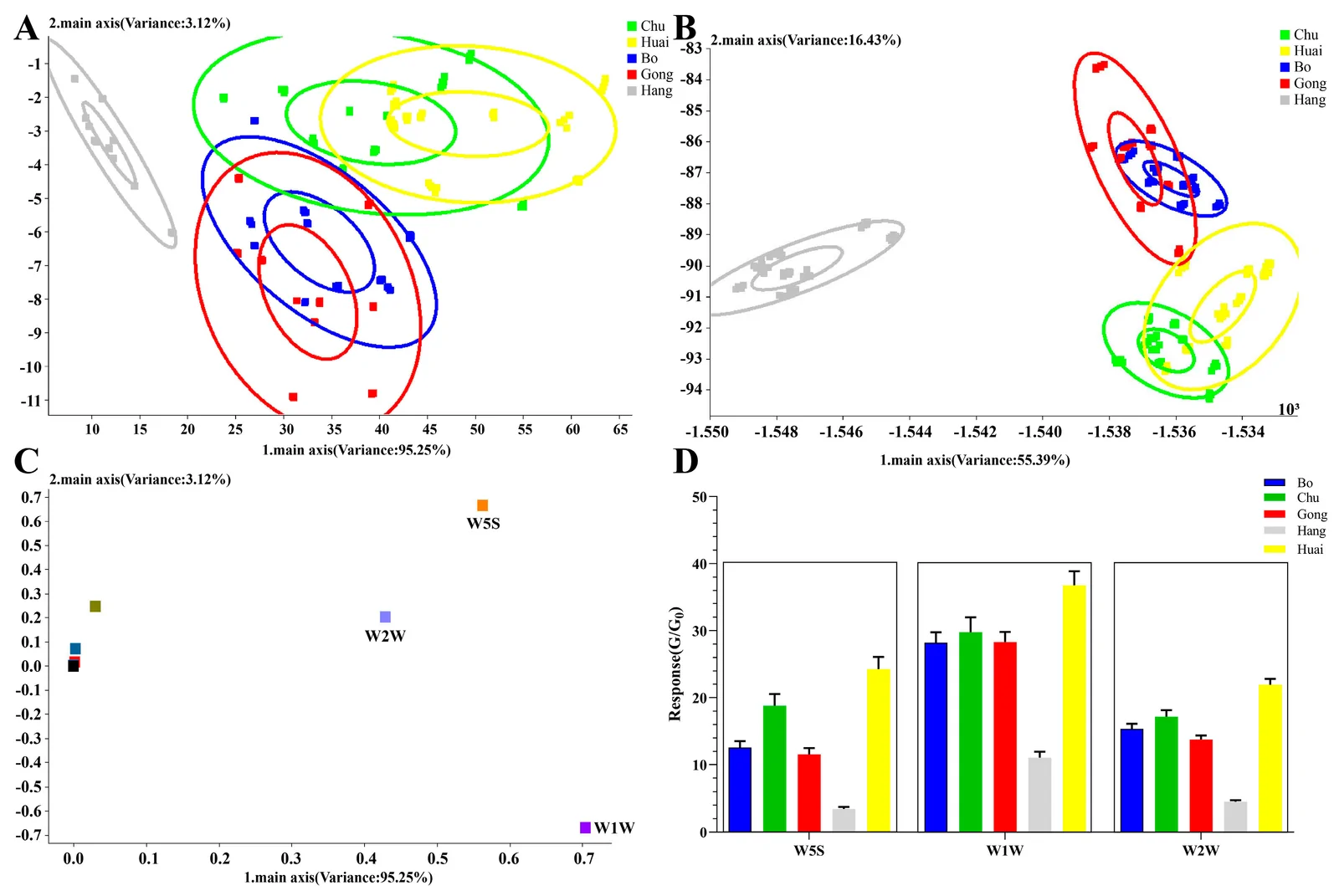
Odor response profiles of five Chrysanthemum varieties analyzed using an electronic nose. (**A**) PCA score plot based on the response signals of electronic nose sensors of five Chrysanthemum varieties. (**B**) LDA score plot showing the discrimination of five Chrysanthemum varieties according to their odor characteristics. (**C**) LOA plot illustrating the contribution of different sensors to sample discrimination. (**D**) Response maps showing the response intensity of electronic nose sensors (W1S, W1W, and W2W) toward different Chrysanthemum varieties. Bo, Boju variety; Chu, Chuju variety; Gong, Gongju variety; Hang, Hangju variety; Huai, Huaiju variety.

The PCA is shown in Figure 5A. PC1 accounted for 95.25% of the total variance, while PC2 explained 3.12% of the variance. It can be seen that except for the Hangju variety, the odor information of the other four Chrysanthemums partially overlapped, indicating that the Hangju variety had a very distinctive odor compared with the other four Chrysanthemums. The odors of the other four Chrysanthemums had similarities as well as their distinctive features. For LDA, the first and second discriminant functions accounted for 55.39% and 16.43% of the original data, respectively. As depicted in Figure 5B, five varieties of Chrysanthemums could be roughly divided into three categories. The Hangju variety was in a separate category, Chuju and Huaiju varieties were in the second category with a more similar odor, and Gongju and Boju varieties were in the third category with a more similar odor. It could be seen that the odor information of Gongju, Chuju, Boju, and Huaiju varieties still partially overlapped, but further separation was obtained compared with the PCA. The results demonstrated that the odors of five varieties of Chrysanthemums have some differences and similarities, and the E-nose possessed some credibility in distinguishing the odors of Chrysanthemums from five different origins.

With LOA, as depicted in Figure 5C, it was determined that certain sensors, namely W1W, W5S, and W2W, made the most significant contributions to the differentiation process of the five varieties, particularly with PC1. Additionally, W5S and W2W were found to have a more meaningful impact on PC2. According to a previous study, W5S, W1W, and W2W sensors are sensitive to nitrogen oxides, sulfur compounds, pyrazines, and aromatic components [28]. Combined with the response characteristics of each sensor, it could be seen that nitrogen oxides, aromatic compounds, and sulfides may be the main factors contributing to the differences in the odors of the five Chrysanthemum varieties.

Furthermore, the histogram displayed the relative intensities of the sensors for Chrysanthemums of five different origins (Figure 5D). The response values of the five Chrysanthemums on the W5S and W2W sensors were in the order of Huaiju > Chuju > Boju > Gongju > Hangju. For the response of W1W, it was in the order of Huaiju > Chuju > Gongju > Boju > Hangju. This revealed that Huaiju had the strongest odor and Hangju had the lightest odor. The response signals of Hangju on the three sensors were much lower than those of the remaining four Chrysanthemums, which may just confirm the phenomenon in Figure 5A. And, previous studies have also shown that Hangju has the lowest content of volatile components [26]. The odors of the other three Chrysanthemums had both similarities and uniqueness, and the comprehensive performance of the composition, types, and content ratios of volatile components constituted the unique flavor of each tea.

### 3.3. Flavor Compounds

#### 3.3.1. Taste Flavor Compounds

##### Results of GRA

**Figure 6:**
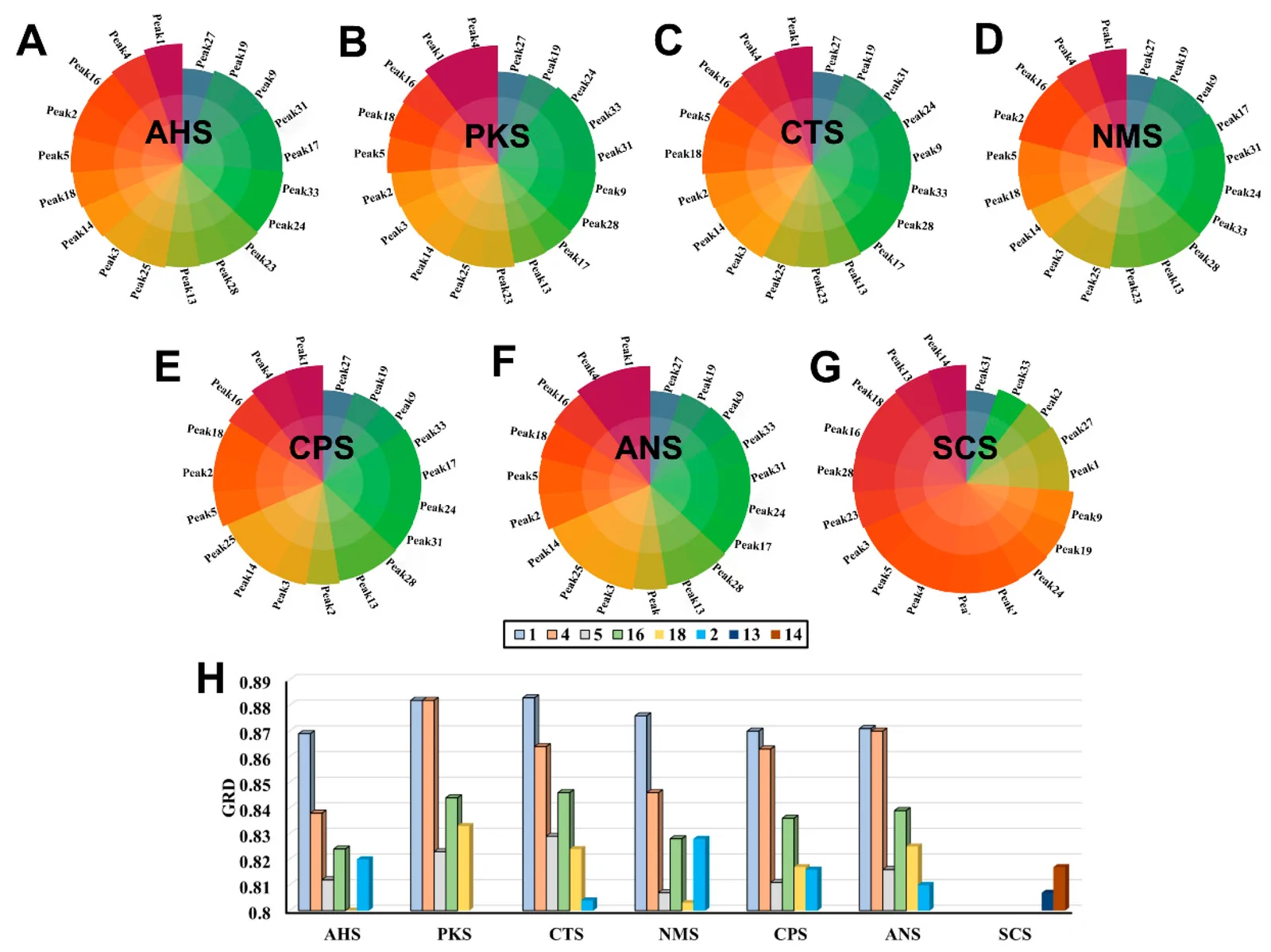
GRA between taste-related compounds and E-tongue sensor responses. (**A**–**G**): Polar plots of gray relational degree (GRD) values between identified taste-related compounds and seven E-tongue sensors based on GRA. (**H**): GRD values of the eight compounds showing strong correlations (GRD > 0.8) with E-tongue sensor responses. The higher GRD value indicates a stronger relationship between the compounds and sensor responses. (AHS, sourness; PKS, the integrated sensor; CTS, saltiness; NMS, umami; CPS, the integrated sensor; ANS, sweet; SCS, bitterness).

The results are presented in polar maps, which depict the calculated and ranked GRD values of the 19 low-molecular-weight chemicals with the seven E-tongue sensors (Figure 6A–G and Table S7). GRD was used to evaluate the contribution of each component to the taste of Chrysanthemums from different origins, and the closer the GRD is to 1, the greater the contribution [29]. Figure 6H demonstrated that certain low-molecular-weight compounds, namely phenylalanine (P1), 1-caffeoylquinic Acid (P2), chlorogenic acid (P4), cryptochlorogenic acid (P5), luteolin-7-*β*-D-glucoside (P13), luteolin-7-*β*-D-glucuronide (P14), 3,4-dicaffeoylquinic acid (P16), and 3,5-dicaffeoylquinic acid (P18), exhibited GRD values greater than 0.8 for the seven sensors. This meant that these specific chemical components provided a significant contribution to the presentation of each flavor.

##### Results of PLSR

**Figure 7:**
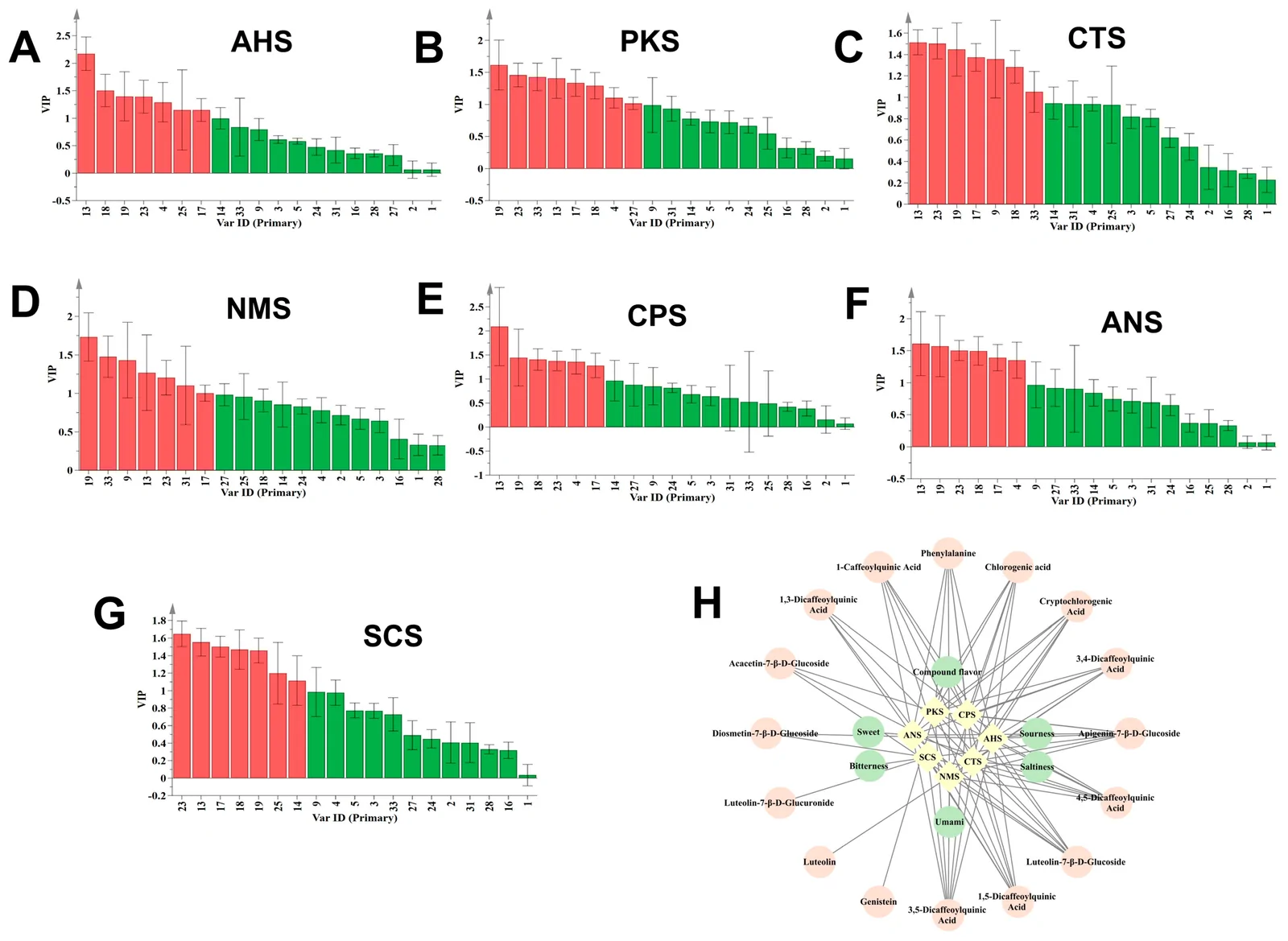
PLSR correlation analysis and visualization network of taste flavor compounds and E-tongue sensors. (**A**–**G**): VIP plot for seven sensors of E-tongue based on PLSR (AHS-SCS). (**H**): Visualization network between taste flavor compounds and E-tongue sensors. (AHS, sourness; PKS, the integrated sensor; CTS, saltiness; NMS, umami; CPS, the integrated sensor; ANS, sweet; SCS, bitterness).

The correlation was assessed by combining the VIP (Figure 7A–G) and regression coefficient (Figure S6 and Table S8) as composite indicators. A positive correlation indicated that an increase in the concentrations of these compounds led to a more pronounced sensor response. The composition of VIP > 1 had a significant effect on the taste of Chrysanthemum [30].

Luteolin-7-O-*β*-D-glucoside (P13) was identified as the most influential variable, followed by P18 > P19 > P23 > P4 > P25 > P17 with the VIP sequence. Additionally, the absolute value of the correlation coefficients sequence ranked P13 > P25 > P4 > P19 > P18 > P23 > P17 in terms of their association with the taste component. It indicated that component (P13) was the primary factor influencing the sourness (AHS sensor) of five kinds of Chrysanthemums, and it was observed that the sourness became stronger with an increase in the content of P13. In contrast, diosmetin-7-*β*-D-glucoside (P25) exhibited a strong negative correlation with sourness. The primary taste components of PKS (the integrated sensor) were chlorogenic acid (P4) and 4,5-dicaffeoylquinic acid (P23), considering comprehensively the VIP sequence of P19 > P23 > P33 > P13 > P17 > P18 > P4 > P27 and the absolute value of correlation coefficient sequence of P4 > P27 > P18 > P13 > P33 > P17 > P23 > P19. The main flavor-presenting compound of saltiness (CTS sensor) was 1,3-dicaffeoylquinic acid (P9), which considering comprehensively the VIP sequence of P13 > P23 > P19 > P17 > P9 > P18 > P33 and the absolute value of correlation coefficient sequence of P9 > P23 > P19 > P13 > P33 > P17 > P18. The primary taste components of umami (NMS sensor) were 1,3-dicaffeoylquinic acid (P9) and apigenin-7-*β*-D-glucoside (P19), which considering comprehensively the VIP sequence of P19 > P33 > P9 > P13 > P23 > P31 > P7 and the absolute value of correlation coefficient sequence of P19 > P9 > P13 > P33 > P23 > P31 > P17. The primary taste constituents of CPS (the integrated sensor) were identified as chlorogenic acid (P4) and luteolin-7-*β*-D-glucoside (P13), considering comprehensively the VIP sequence of P13 > P19 > P18 > P23 > P4 > P17 and the absolute value of correlation coefficient sequence of P4 > P13 > P18 > P23 > P17 > P19. Similarly, the primary flavor component of bitterness (SCS sensor) was diosmetin-7-*β*-D-glucoside (P25), which had the largest absolute value of correlation coefficient and VIP > 1. For sweet (ANS sensor), the main taste constituents were chlorogenic acid (P4), 3,5-dicaffeoylquinic acid (P18), and 4,5-dicaffeoylquinic acid (P23). These results were similar to those of GRA and further provided a more comprehensive understanding.

The construction of the sensor–taste flavor compounds–flavor network is depicted in Figure 7H. The network has 83 edges and 32 nodes, including seven sensors, 16 compounds, and six odor characteristics. The pink circles symbolize the ultimate potential taste flavor compounds for five kinds of Chrysanthemums, and the top 10 in order of degree were 3,5-dicaffeoylquinic acid (P18), 1,5-dicaffeoylquinic acid (P17), luteolin-7-*β*-D-glucoside (P13), 4,5-dicaffeoylquinic acid (P23), apigenin-7-*β*-D-glucoside (P19), 3,4-dicaffeoylquinic acid (P16), cryptochlorogenic acid (P5), chlorogenic acid (P4), phenylalanine (P1), and 1-caffeoylquinic acid (P2). Chengyu Gao et al. discovered that 4,5-dicaffeoylquinic acid (P23), 4-caffeoylquinic acid, and 5-caffeoylquinic acid in coffee could reduce the perception of bitterness in brewed coffee [31], meaning that they have the potential to influence the Chrysanthemums’ flavor. And, 1,5-caffeoylquinic acid and its derivatives, including 4,5-dicaffeoylquinic acid (P23), created a mildly astringent perception [32]. Chlorogenic acid (P4) was identified as a bitter and astringent compound that has a sour and astringent taste and a synergistic effect with catechins on the bitter taste of tea in past studies [33,34] Chlorogenic acid (P4) was easily degraded into caffeic acid and quinic acid, which affect the bitterness of coffee and were proposed as key mechanisms of flavor instability [35,36]. Previous studies have also shown that phenylalanine (P1) played a part in the bitter taste in oolong tea infusion, and that was identified as an irritant [37,38]. These results coincided with our findings that these taste flavor compounds were closely related to the taste of Chrysanthemums.

These flavor compounds are the primary cause of the differences in taste among the five kinds of Chrysanthemums and are the reason that Hangju is the strongest in sweetness, bitterness, saltiness, and complex flavor and Hangju is loved by consumers as a tea in the market.

#### 3.3.2. Volatile Flavor Compounds

**Figure 8:**
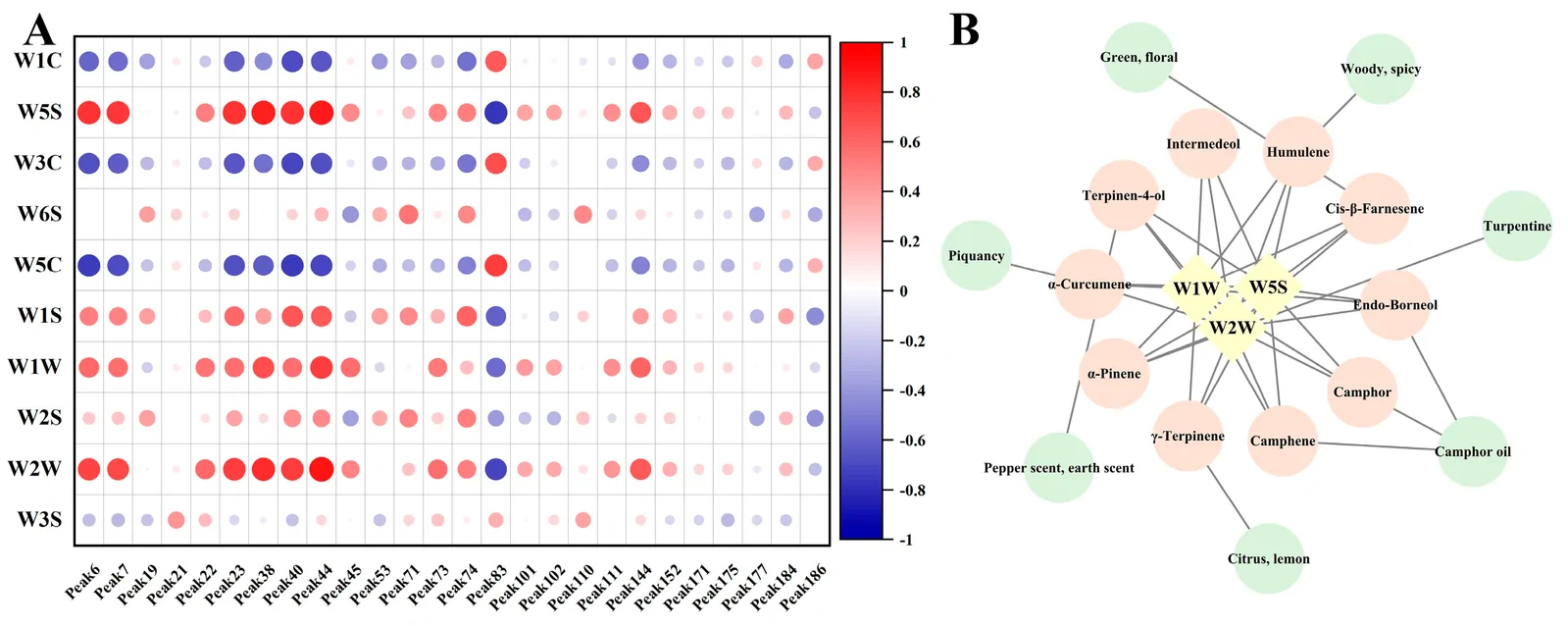
Spearman correlation analysis and visualization network between volatile flavor compounds and electronic nose sensor responses. (**A**) Heatmap showing the Spearman correlation coefficients between volatile compounds and electronic nose sensors. The color gradient indicates the correlation strength, with red and blue indicating positive and negative correlations, respectively. (**B**) Correlation network illustrating the relationships between key volatile compounds and electronic nose sensors. The nodes represent volatile compounds, sensors, and odor characteristics, and the connecting lines indicate significant correlation.

As shown in Figure 8A and Table S9, it was found that there were obvious differences between the three sensors W5S, W2W, and W1W based on the comprehensive analysis of the E-nose detection results. Odor profiles of volatile compounds can be found at http://www.leffingwell.com (accessed on 16 September 2026). The results showed that *α*-pinene (P6), camphene (P7), *γ*-terpinene (P23), camphor (P38), endo-borneol (P40), terpinen-4-ol (P44), humulene (P73), cis-*β*-farnesene (P74), and intermedeol (P144) had strong positive associations between the response values of W5S, W2W, and W1W, while *α*-curcumene (P83) demonstrated a negative correlation. Combining the aroma characteristics of the odor-related substances (Table S9) and the response strengths of the five varieties of Chrysanthemums on three sensors, we knew that Huaiju had the strongest pine, woody, pungent, camphoraceous, floral, and fruity aroma, followed by Chuju > Boju > Gongju, and Hangju had the weakest aroma. *α*-pinene (P6), camphene (P7), and camphor (P38) were identified as the characteristic aroma compounds by odor activity value (OAV) from the unique aroma of chrysanthemum nankingense [12].

Sun et al. reported that monoterpenoids and oxygenated monoterpenoids accounted for a major proportion of floral volatiles in Chrysanthemum variety, with camphor, α-pinene, and camphene identified as important volatile compounds [39]. The correlation analysis of GC-MS and E-nose showed that *α*-pinene (P6) and camphor (P38) were significantly related to the sensors of E-nose. These results validated our results and helped to further understand the flavor of chrysanthemum. Li et al. combined GC-O, GC-MS, and multivariate statistical analysis to identify aroma-active compounds in Chrysanthemum essential oils and found that α-pinene, camphor, camphene, caryophyllene, and (Z)-β-farnesene were closely associated with characteristic aroma attributes, including floral and woody notes [5]. Consistent with these previous findings, α-pinene, camphor, terpinen-4-ol, humulene, and cis-β-farnesene identified in the present study showed strong correlations with electronic nose responses, suggesting their important contribution to odor differentiation among the five Chrysanthemum varieties. Furthermore, previous studies have indicated the volatile compounds of Chrysanthemum varies among different varieties, which may result in distinct aroma profiles. Therefore, the stronger odor-associated responses observed for Huaiju may be attributed to differences in the composition and relative abundance of characteristic volatile compounds.

The network diagram (Figure 8B) illustrated a system comprising 39 edges and 20 nodes, including three sensors, 10 compounds, and seven odor characteristics. Notably, the prominent volatile flavor compounds were represented by the pink circles. That may be the reason why the Huaiju variety has more turpentine, citrus, camphor oil, pepper scent, woody, green, and piquancy and the Huaiju variety is obviously different from that of the other four Chrysanthemums.

### 3.4. Limitations and Future Perspectives

In this study, multiple independent batches of each Chrysanthemum variety were analyzed to characterize their chemical and sensory profiles. Because sample collection was conducted within a single production period, further investigations across consecutive years are needed to evaluate the temporal stability of the identified characteristic compounds and sensory patterns. In addition, the identification of compounds through LC-Q-TOF-MS and GC-MS was mainly based on accurate mass, MS/MS fragmentation information, and database matching. Some representative compounds were further confirmed using authentic standards; however, additional standard-based validation and targeted quantitative analysis of more compounds would further enhance the reliability of compound identification and quantitative assessment. The relative abundance of volatile compounds obtained through GC-MS provided valuable comparative information, while quantitative analysis with internal standards could further improve the accuracy of contribution assessment. Moreover, although E-nose, E-tongue, and chemometric approaches (GRA, PLSR, and Spearman correlation analysis) effectively revealed associations between chemical constituents and sensory characteristics, further sensory evaluation and targeted validation are required to clarify the specific contributions of individual compounds.

Despite these considerations, the integrated strategy combining LC-Q-TOF-MS, GC-MS, electronic nose, electronic tongue, and chemometric approaches provides a comprehensive approach for understanding the relationship between chemical composition and sensory properties of Chrysanthemum varieties and offers potential markers for quality evaluation and variety identification.

## 4. Conclusions

This study established an integrated analytical strategy combining E-tongue, E-nose, LC-Q-TOF-MS, GC-MS, and chemometric approaches to distinguish five varieties of Chrysanthemums. Characteristic chemical profiles of the five Chrysanthemum varieties were revealed based on their low molecular fraction and volatile fraction compounds. The 19 low molecule fractions compounds and 26 volatile fractions compounds from five varieties of Chrysanthemums were examined using LC-Q-TOF-MS and GC-MS. Potential characteristic compounds contributing to discrimination among five varieties of Chrysanthemums were screened through chemometrics, which contained the Luteolin-7-*β*-D-glucoside, apigenin-7-*β*-D-glucoside, 4,5-dicaffeoylquinic acid, *α*-terpineol, terpinen-4-ol, camphor, etc. The taste and odor of five varieties of Chrysanthemums were rapidly distinguished using E-tongue and E-nose. Hangju had the richest taste flavor and the weakest arom and Huaiju had the strongest odor of pine, woody, floral, and fruity aroma. Finally, GRA and PLSR results showed that 3,5-dicaffeoylquinic acid, 1,5-dicaffeoylquinic acid, luteolin-7-*β*-D-glucoside, 4,5-dicaffeoylquinic acid, apigenin-7-*β*-D-glucoside, 3,4-dicaffeoylquinic acid, etc. were considered the taste flavor compounds of five varieties of Chrysanthemums. The primary volatile flavor compounds were identified through Spearman analysis, which were *α*-pinene, camphene, *γ*-terpinene, camphor, endo-borneol, terpinen-4-ol, humulene, cis-*β*-farnesene, and intermedeol. The study successfully distinguished the material composition, odor, and taste of five varieties of Chrysanthemums. This finding holds significant implications for the rational utilization and flavor chemistry of Chrysanthemum resources, providing a solid scientific foundation.

## Data Availability

The original contributions presented in this study are included in the article/Supplementary Material. Further inquiries can be directed to the corresponding authors.

## Supplementary Materials

The following supporting information can be downloaded at: https://www.mdpi.com/article/10.3390/foods15183328/s1.
