# Supplementary material for: Comparative Study on Taste and Odor Flavor Compounds of Chrysanthemums from Five Different Varieties Using UPLC-Q-TOF-MS, GC-MS, E-Tongue, E-Nose, and Chemometrics
Source: Foods. 2026 Sep 19;15(18):3328. doi: 10.3390/foods15183328 (PMC13606529; doi:10.3390/foods15183328)
Supplement: Supplementary file 1 [file foods-15-03328-s001.zip › foods-4526629-supplementary.pdf]

Supplementary information

Table S1. 50 batches of information on Chrysanthemums.

Table S2. The performance description of PEN3.5 E-nose.

Table S3. HPLC-Q-TOF-MS information of low molecule fractions compounds from five kinds of Chrysanthemums.

Table S4. GC-MS information of the volatile fractions compounds from five kinds of Chrysanthemums.

Table S5. HPLC fingerprint similarity information of the low molecule fractions compounds.

Table S6. GC fingerprint similarity information of the volatile fractions compounds.

Table S7. GRD of E-tongue and common peaks of the low molecule fractions compounds.

Table S8. Regression coefficients of E-tongue and common peaks of the low molecule fractions compounds.

Table-S9. Spearman correlation analysis of E-nose and common peaks of the volatile fractions compounds.

Fig. S1. Traditional geographical origins of the five varieties of Chrysanthemum.

Fig. S2. Total ion chromatograms (TIC) of the low molecule fractions components of five kinds of Chrysanthemums obtained by LC–Q–TOF–MS analysis. (A. Boju-positive ion mode; B. Boju-negative ion mode; C. Chuju-positive ion mode; D. Chuju-negative ion mode; E. Gongju-positive ion mode; F. Gongju-negative ion mode; G. Hangju-Chrysanthemum positive ion mode; H. Hangju-Chrysanthemum negative ion mode; I. Huaijiu-positive ion mode; J. Huaijiu-negative ion mode.).

Fig. S3. Identification of the volatile fraction compounds of five kinds of Chrysanthemums obtained by GC–MS analysis. A. Boju; B. Chuju; C. Gongju; D. Hangju; E. Huaijiu.

Fig. S4. Types of the volatile fractions compounds for Chrysanthemums (A) and five kinds of Chrysanthemums (B).

Fig. S5. Response curve of 10 sensors of E-nose.

Fig. S6. Regression coefficient plots of seven sensors for E-tongue based on PLSR. (A): AHS; (B): PKS; (C): CTS; (D): NMS; (E): CPS; (F): ANS; (G): SCS.

Table-S1. 50 batches of information on Chrysanthemums.

No.	Category	Origins	No.	Category	Origins	No.	Category	Origins	No.	Category	Origins	No.	Category	Origins
Bo-1	Boju	Bozhou	Chu-1	Chuju	Chuzhou	Gong-1	Gongju	Huangshan	Hang-1	Hangju	Tongxiang	Huai-1	Huaiju	Jiaozuo
Bo-2	Boju	Bozhou	Chu-2	Chuju	Chuzhou	Gong-2	Gongju	Huangshan	Hang-2	Hangju	Tongxiang	Huai-2	Huaiju	Jiaozuo
Bo-3	Boju	Bozhou	Chu-3	Chuju	Chuzhou	Gong-3	Gongju	Huangshan	Hang-3	Hangju	Tongxiang	Huai-3	Huaiju	Jiaozuo
Bo-4	Boju	Bozhou	Chu-4	Chuju	Chuzhou	Gong-4	Gongju	Huangshan	Hang-4	Hangju	Tongxiang	Huai-4	Huaiju	Jiaozuo
Bo-5	Boju	Bozhou	Chu-5	Chuju	Chuzhou	Gong-5	Gongju	Huangshan	Hang-5	Hangju	Tongxiang	Huai-5	Huaiju	Jiaozuo
Bo-6	Boju	Bozhou	Chu-6	Chuju	Chuzhou	Gong-6	Gongju	Huangshan	Hang-6	Hangju	Tongxiang	Huai-6	Huaiju	Jiaozuo
Bo-7	Boju	Bozhou	Chu-7	Chuju	Chuzhou	Gong-7	Gongju	Huangshan	Hang-7	Hangju	Tongxiang	Huai-7	Huaiju	Jiaozuo
Bo-8	Boju	Bozhou	Chu-8	Chuju	Chuzhou	Gong-8	Gongju	Huangshan	Hang-8	Hangju	Tongxiang	Huai-8	Huaiju	Jiaozuo
Bo-9	Boju	Bozhou	Chu-9	Chuju	Chuzhou	Gong-9	Gongju	Huangshan	Hang-9	Hangju	Tongxiang	Huai-9	Huaiju	Jiaozuo
Bo-10	Boju	Bozhou	Chu-10	Chuju	Chuzhou	Gong-10	Gongju	Huangshan	Hang-10	Hangju	Tongxiang	Huai-10	Huaiju	Jiaozuo

Table-S2. The performance description of PEN 3.5 E-nose.

NO.	Sensor name	Performance description
S1	W1C	Aromatic constituents, benzene
S2	W5S	High sensitivity and sensitive to nitrogen oxides
S3	W3C	Sensitive aroma, ammonia
S4	W6S	Mainly selective for hydrides
S5	W5C	Short-chain alkane aromatic component
S6	W1S	Sensitive to methyl
S7	W1W	Sensitive to sulfides
S8	W2S	Sensitive to alcohols, aldehydes and ketones
S9	W2W	Aromatic ingredients, sensitive to organic sulfides
S10	W3S	Sensitive to long-chain alkanes

Table-S3. HPLC-Q-TOF-MS information of low molecule fractions compounds from five kinds of Chrysanthemums.

NO.	tR(min)	M+H/M-H(m/z)	Formula	CAS	Fragments(m/z)	Compounds	Bo	Chu	Gong	Hang	Huai
1	5.63	/164.0705	$C_{10}H_{13}NO_2$	673-31-4	164.0711,147.0432,103.0528	Phenylalanine	+	+	+	+	+
2	7.71	/353.0877	$C_{16}H_{18}O_9$	1241-87-8	135.0465,179.0316,191.0548	**1-Caffeoylquinic Acid (Std)**	+	+	+	+	+
3	9.21	/353.0878	$C_{16}H_{18}O_9$	906-33-2	135.0448,179.0343,191.0550	**Neochlorogenic Acid (Std)**	+	+	+	+	+
4	12.94	/353.0880	$C_{16}H_{18}O_9$	327-97-9	135.0432,179.0349,191.0553	**Chlorogenic acid (Std)**	+	+	+	+	+
5	13.52	/353.0881	$C_{16}H_{18}O_9$	905-99-7	173.0449,179.0340	**Cryptochlorogenic Acid (Std)**	+	+	+	+	+
6	15.59	/431.1910	$C_{21}H_{20}O_{10}$	578-74-5	269.078	Apigenin-7-β-D-galactoside	-	+	+	+	-
7	15.89	/179.0342	$C_9H_8O_4$	331-39-5	135.0443	Caffeic Acid	+	+	+	+	+
8	16.33	/593.1510	$C_{27}H_{30}O_{15}$	20633-84-5	283.0614,297.0728	Luteolin-7-β-D-rutinoside	+	-	+	+	+
9	17.86	/515.1180	$C_{25}H_{24}O_{12}$	30964-13-7	191.0551,353.0869,179.0337	**1,3-Dicaffeoylquinic Acid(Std)**	+	+	+	+	+
10	18.28	/609.1433	$C_{27}H_{30}O_{16}$	153-18-4	607.3716,608.1907,610.1486	Rutin	+	+	+	-	+
11	22.27	/609.1444	$C_{28}H_{34}O_{15}$	520-26-3	610.1462,611.1453	Hesperidin	+	+	+	-	+
12	26.73	/449.1066	$C_{21}H_{22}O_{11}$	38965-51-4	287.0544,151.0024	Eriodictyol-7-β-D-Glucoside	+	+	+	+	+
13	29.8	/447.0922	$C_{21}H_{20}O_{11}$	5373/11/5	285.0392,151.0021,256.0380	Luteolin-7-β-D-Glucoside	+	+	+	+	+
14	31.15	/461.0708	$C_{21}H_{18}O_{12}$	29741-10-4	285.0391,151.0031	Luteolin-7-β-D-Glucuronide	+	+	+	+	+
15	32.41	/515.1134	$C_{25}H_{24}O_{12}$	1182-34-9	353.0876,179.0312,173.0433	**1,4-Dicaffeoylquinic Acid (Std)**	+	+	-	+	+
16	34.28	/515.1189	$C_{25}H_{24}O_{12}$	57378-72-0	353.0865,203.0335,173.0445	**3,4-Dicaffeoylquinic Acid (Std)**	+	+	+	+	+
17	35.23	/515.1193	$C_{25}H_{24}O_{12}$	30964-13-7	353.0881,173.0450	**1,5-Dicaffeoylquinic Acid (Std)**	+	+	+	+	+
18	37.89	/515.1188	$C_{25}H_{24}O_{12}$	89919-62-0	353.0876,191.0551,179.0337	**3,5-Dicaffeoylquinic Acid (Std)**	+	+	+	+	+
19	41.42	/431.0981	$C_{21}H_{20}O_{10}$	578-74-5	268.0366,269.0442	Apigenin-7-β-D-Glucoside	+	+	+	+	+

20	41.91	/411.5219	$C_{22}H_{36}O_7$	4720-09-6	344.9762,119.0865	grayanotoxin I	+	-	-	+	+
21	41.98	/577.1542	$C_{27}H_{30}O_{14}$	552-57-8	269.0438	Apigenin-7-O-rutinoside	-	+	-	+	+
22	43.76	/445.0770	$C_{21}H_{18}O_{11}$	29741-09-1	269.0447,113.0242,175.0217	Apigenin-7-β-D-Glucuronide	+	+	-	+	+
23	44.91	/515.1185	$C_{25}H_{24}O_{12}$	57378-72-0	173.0442,353.0869,191.0552	4,5-Dicaffeoylquinic Acid	+	+	+	+	+
24	46.16	/533.0930	$C_{24}H_{22}O_{14}$	98767-38-5	285.0397	Luteolin-7-β-D-(6″-O-malonyl)-glucoside	+	+	+	+	+
25	46.9	463.1225/	$C_{22}H_{22}O_{11}$	20126-59-4	301.0696	Diosmetin-7-β-D-Glucoside	+	+	+	+	+
26	49.04	477.1014/	$C_{22}H_{20}O_{12}$	35110-20-4	301.0710,302.0751,286.0478	Diosmetin-7-β-D-Glucuronide	+	+	-	+	+
27	56.88	/269.0446	$C_{15}H_{10}O_5$	446-72-0	159.0440,135.0442	Genistein	+	+	+	+	+
28	57.29	/293.1758	$C_{17}H_{26}O_4$	131119-10-3	293.1725,219.04841	(4-hydroxy-3-methoxyphenyl) methylnonanoate	+	+	+	+	+
29	58.47	/547.1088	$C_{21}H_{22}O_{10}$	529-55-5	299.0567	Diosmetin-7-β-D-(6″-O-malonyl)-glucoside	+	+	-	+	+
30	59.82	593.1886/	$C_{28}H_{32}O_{14}$	480-36-4	285.0766,447.1272,593.1892	Buddleoside	+	-	+	+	+
31	60.52	/285.0399	$C_{15}H_{10}O_6$	491-70-3	133.0285,151.0042,175.0396	Luteolin	+	+	+	+	+
32	61.67	/473.1063	$C_{23}H_{22}O_{11}$	72741-92-5	268.0359,269.0421	Apigenin-7-β-D-(6″-O-acetyl)-Glucoside	+	+	+	+	-
33	62.05	/445.1100	$C_{22}H_{22}O_{10}$	4291-60-5	283.0604	Acacetin-7-β-D-Glucoside	+	+	+	+	+
34	63.03	/459.0921	$C_{22}H_{20}O_{11}$	38226-83-4	283.0592	Acacetin-7-β-D-Glucuronide	+	+	-	+	+
35	65.09	/271.0603	$C_{15}H_{12}O_5$	480-41-1	151.0027,119.0493	Naringenin	+	-	-	-	+
36	65.86	/301.0703	$C_{15}H_{10}O_7$	117-39-5	151.0032,121.7481	Quercetin	-	+	-	-	-
37	66.04	/269.0447	$C_{15}H_{10}O5$	520-36-5	117.0331,118.0369,107.0135	Apigenin	+	+	+	+	+

					121.0276,151.0033					
38	67.29	/299.0561	$C_{16}H_{12}O_6$	520-34-3	299.0556,300.0590,284.0324 285.0364,301.0639	Diosmetin	+	+	-	-	+
39	69.83	/287.2222	$C_{15}H_{12}O_6$	552-58-9	287.2226	Eriodictyol	+	+	+	-	+
40	73.4	/373.0926	$C_{19}H_{18}O_8$	111537-41-8	343.0476	3,5-Dihydroxy-4',6,7,8-tetramethoxyflavone	-	-	+	-	-
41	74.05	/343.0814	$C_{18}H_{16}O_7$	22368-21-4	328.0600,343.0829,313.0314 329.0609	Eupatilin	+	-	+	-	-
42	78.65	/283.0611	$C_{16}H_{12}O_5$	480-44-4	151.0013,117.0333	Acacetin	+	+	-	-	+

Std: confirmed with standards.

Table-S4. GC-MS information of the volatile fractions compounds from five kinds of Chrysanthemums.

NO.	RT (min)	Compound	Formula	Relative content (%)					CAS	Classes
				Bo	Chu	Gong	Hang	Huai		
1	5.36	Furfural	$C_5H_4O_2$	-	0.72	-	-	-	98-01-1	Aldehydes
2	5.39	1,3-Cyclopentadiene,5,5-dimethyl-1-ethyl-	C_9H_{14}	0.96	-	-	-	-	496862-86-3	Olefins
3	5.41	Unknown	$C_{13}H_{14}O_2$	-	-	-	0.86	-		
4	6.04	2-Hexenal, (E)-	$C_6H_{10}O$	-	-	0.09	-	-	6728-26-3	Aldehydes
5	8.41	Tricyclene	$C_{10}H_{16}$	-	-	-	-	0.06	112-31-2	Terpenoids
6	8.85	α-Pinene	$C_{10}H_{16}$	0.82	0.73	0.75	0.11	0.41	80-56-8	Olefins
7	9.37	Camphene	$C_{10}H_{16}$	0.1	1.04	0.24	0.16	1.4	79-92-5	Olefins
8	10.28	2-Cyclopenten-1-one,2,3,5-trimethyl-4-methylene-	$C_9H_{12}O$	-	0.72	-	-	-	29765-85-3	Ketones
9	10.29	β-Phellandrene	$C_{10}H_{16}$	-	-	-	-	0.15	555-10-2	Olefins
10	10.3	β-Terpinen	$C_{10}H_{16}$	-	-	0.17	-	-	99-86-5	Olefins
11	10.38	β-Pinene	$C_{10}H_{16}$	0.3	0.45	-	-	0.16	127-91-3	Olefins
12	10.56	1-Octen-3-ol	$C_8H_{16}O$	-	-	0.14	-	-	3391-86-4	Alcohols
13	10.9	2,3-Dehydro-1,8-cineole	$C_{10}H_{16}O$	-	-	-	0.34	0.1	92760-25-3	Olefins
14	10.95	β-Myrcene	$C_{10}H_{16}$	-	0.2	-	-	0.07	123-35-3	Olefins
15	10.97	1,6-Dimethylhepta-1,3,5-triene	C_9H_{14}	0.79	-	-	-	-	928-67-6	Olefins
16	10.98	Furan,2-pentyl-	$C_9H_{14}O$	-	-	0.16	-	-	64079-01-2	Ether
17	11.38	α-Phellandrene	$C_{10}H_{16}$	-	-	0.21	-	-	99-83-2	Olefins
18	11.83	Isoterpinolene	$C_{10}H_{16}$	-	0.76	0.11	0.4	0.15	43124-56-7	Olefins
19	12.14	o-Cymene	$C_{10}H_{14}$	0.4	0.37	0.78	0.42	0.1	527-84-4	Aromatic compounds
20	12.21	2-Caren-4-ol	$C_{10}H_{16}O$	-	-	-	0.4	-	6617-35-2	Alcohols
21	12.38	Eucalyptol	$C_{10}H_{18}O$	3.19	15.73	2.79	0.2	3.4	470-82-6	Alcohols
22	12.89	Benzeneacetaldehyde	C_8H_8O	0.51	0.35	0.16	0.24	0.08	122-78-1	Aldehydes
23	13.52	γ-Terpinene	$C_{10}H_{16}$	0.34	1.28	0.21	0.79	0.25	99-85-4	Olefins

24	13.87	Bicyclo[3.1.0]hexan-2-ol,2-methyl-5-(1-methylethyl)-,(1α,2α,5α)-	$C_{10}H_{18}O$	-	-	-	0.14	-	17699-16-0	Alcohols
25	14.78	Terpinolene	$C_{10}H_{16}$	-	0.29	-	0.25	-	586-62-9	Olefins
26	15.22	Bicyclo[3.1.0]hexan-2-ol,2-methyl-5-(1-methylethyl)-,(1α,2β,5α)-	$C_{10}H_{18}O$	-	-	-	0.27	0.08	15537-55-0	Alcohols
27	15.23	Unknown	$C_{10}H_{18}O$	-	0.21	-	-	-		
28	15.48	Filifolone	$C_{10}H_{14}O$	3.67	-	-	-	-	16750-87-1	Ketones
29	15.55	Phellandral	$C_{10}H_{16}O$	-	0.22	-	-	-	21391-98-0	Aldehydes
30	15.59	ε-Cyclogeraniolene	C_9H_{16}	-	-	-	-	0.18	503-45-7	Olefins
31	15.96	1-Octen-3-yl-acetate	$C_{10}H_{18}O_2$	-	0.18	-	-	-	2442-10-6	Esters
32	16	trans-Chrysanthenol	$C_{10}H_{16}O$	0.47	-	4.96	-	-	38043-83-3	Alcohols
33	16.27	2-Cyclohexen-1-ol,1-methyl-4-(1-methylethenyl)-, trans-	$C_{10}H_{16}O$	-	-	-	0.27	-	7212-40-0	Alcohols
34	16.29	cis-2-p-Menthen-1-ol	$C_{10}H_{18}O$	-	0.16	-	-	-	29803-82-5	Alcohols
35	16.58	Chrysanthenone	$C_{10}H_{14}O$	21	-	-	0.28	-	473-06-3	Ketones
36	17.01	Isocyclocitral	$C_{10}H_{16}O$	1.14	-	0.47	0.98	-	1335-66-6	Aldehydes
37	17.22	L-trans-Pinocarveol	$C_{10}H_{16}O$	-	-	-	-	0.21	547-61-5	Alcohols
38	17.36	Camphor	$C_{10}H_{16}O$	2.05	15.46	2.75	0.6	11.52	21368-68-3	Ketones
39	18.29	Pinocarvone	$C_{10}H_{14}O$	-	-	-	-	0.21	34-41-3	Ketones
40	18.39	endo-Borneol	$C_{10}H_{18}O$	4.61	1.5	1.64	0.67	17.33	507-70-0	Alcohols
41	18.53	Terpineol	$C_{10}H_{18}O$	-	0.41	-	-	-	8006-39-1	Alcohols
42	18.57	Verbenol	$C_{10}H_{16}O$	0.5	-	-	-	-	473-67-6	Alcohols
43	18.86	3-Thujen-2-one	$C_{10}H_{14}O$	0.26	-	-	0.16	-	24545-81-1	Ketones
44	19.07	Terpinen-4-ol	$C_{10}H_{18}O$	0.78	2.74	0.4	1.48	0.57	562-74-3	Alcohols
45	19.77	α-Terpineol	$C_{10}H_{18}O$	0.94	2.11	0.12	0.3	0.36	98-55-5	Alcohols
46	20.05	(-)-Myrtenol	$C_{10}H_{16}O$	-	-	-	-	0.28	19894-97-4	Alcohols
47	20.84	Unknown	$C_{10}H_{16}O$	1.22	-	-	-	-		
48	22.28	trans-Chrysanthenyl acetate	$C_{12}H_{18}O_2$	2.24	0.17	36.34	0.77	-	54324-99-1	Esters
49	22.58	(E)-Ocimenone	$C_{10}H_{14}O$	0.3	-	-	-	-	33746-72-4	Ketones

50	23.99	cis-Chrysanthenyl acetate	$C_{12}H_{18}O_2$	8.37	-	-	-	-	67999-48-8	Esters
51	24.55	2,4-Dimethylphenethyl alcohol	$C_{10}H_{14}O$	-	0.17	-	-	-	6597-59-7	Alcohols
52	24.57	Isopiperitenone	$C_{10}H_{14}O$	0.72	-	-	-	-	529-01-1	Ketones
53	25.69	(-)-Bornyl acetate	$C_{12}H_{20}O_2$	0.14	2.06	3.39	0.31	2.35	5655-61-8	Esters
54	26.64	Thymol	$C_{10}H_{14}O$	0.26	-	-	0.34	-	89-83-8	Alcohols
55	27.58	2-Propenal,3-(2,2,6-trimethyl-7-oxabicyclo [4.1.0] hept-1-yl)-	$C_{12}H_{18}O_2$	1.66	-	-	-	-	55759-91-6	Aldehydes
56	28.16	Unknown	$C_{11}H_{18}N_2$	0.49	-	-	-	-		
57	28.17	δ-Terpineol acetate	$C_{12}H_{20}O_2$	-	0.47	-	-	-	93836-50-1	Esters
58	29.86	cis-Carvyl acetate	$C_{12}H_{18}O_2$	-	0.2	-	-	-	1205-42-1	Esters
59	30.66	α-Terpinyl acetate	$C_{12}H_{20}O_2$	-	3.22	-	-	-	80-26-2	Esters
60	31.21	Eugenol	$C_{10}H_{12}O_2$	-	-	-	-	0.19	97-53-0	Alcohols
61	31.22	cis-Isoeugenol	$C_{10}H_{12}O_2$	0.33	-	-	-	-	5912-86-7	Alcohols
62	31.99	(+)-trans-Chrysanthemic acid	$C_{10}H_{16}O_2$	2.94	-	-	-	-	4638-92-0	Carboxylic acids
63	32.36	Copaene	$C_{15}H_{24}$	-	0.22	-	0.25	-	3856-25-5	Olefins
64	32.98	(-)-α-Isocomene	$C_{15}H_{24}$	-	-	-	0.5	-	65372-78-3	Olefins
65	33.45	Unknown	$C_{13}H_{20}$	-	0.74	-	-	-		
66	33.52	Safranal	$C_{10}H_{14}O$	0.32	-	-	-	-	116-26-7	Aldehydes
67	33.56	(-)-β-Elemene	$C_{15}H_{24}$	-	0.25	-	0.14	0.27	515-13-9	Olefins
68	34.05	4,7,7-Trimethylbicyclo [4.1.0] hept-3-en-2-one	$C_{10}H_{14}O$	3.92	-	-	-	-	81800-50-2	Ketones
69	34.37	(±)-Thujpsadiene	$C_{15}H_{22}$	-	0.19	-	-	-	51446-91-4	Olefins
70	34.6	Benzenebutanal, γ,4-dimethyl-	$C_{12}H_{16}O$	-	0.39	-	-	-	4895-19-6	Aldehydes
71	35.23	β-Caryophyllene	$C_{15}H_{24}$	2.26	2.93	0.93	3.26	0.53	87-44-5	Olefins
72	36.52	cis-α-Bergamotene	$C_{15}H_{24}$	0.27	-	0.3	-	-	18252-46-5	Olefins
73	37.45	Humulene	$C_{15}H_{24}$	0.25	0.45	0.12	0.3	0.04	6753-98-6	Olefins
74	38.15	cis-β-Farnesene	$C_{15}H_{24}$	1.11	3.31	2.36	4.26	1.04	28973-97-9	Olefins
75	38.57	4,5-di-epi-aristolochene	$C_{15}H_{24}$	-	-	-	0.65	-	54868-40-5	Olefins
76	38.8	(1R,3S,4S)-1,3-Dimethyl-3-(4-	$C_{15}H_{24}O$	-	-	-	0.58	0.67	211237-38-6	Olefins

		methylpent-3-en-1-yl)-2-oxabicyclo [2.2.2] oct-5-ene								
77	39.06	epi-γ-Eudesmol	$C_{15}H_{26}O$	-	-	-	0.69	-	117066-77-0	Alcohols
78	39.37	β-Copaene	$C_{15}H_{24}$	0.43	-	-	6.27	-	18252-44-3	Olefins
79	39.38	cis-muurola-4(14),5-diene	$C_{15}H_{24}$	-	2.98	0.53	-	-	157477-72-0	Olefins
80	39.53	γ-Curcumene	$C_{15}H_{24}$	-	0.38	-	-	-	28976-68-3	Olefins
81	39.54	2-Isopropenyl-4a,8-dimethyl-1,2,3,4,4a,5,6,7-octahydronaphthalene	$C_{15}H_{24}$	0.86	-	0.21	-	-	103827-22-1	Olefins
82	39.56	Isocaryophyllene	$C_{15}H_{24}$	-	-	-	13.94	-	87-44-5	Olefins
83	39.8	α-Curcumene	$C_{15}H_{22}$	2.81	2.81	6.5	4.49	4.81	644-30-4	Olefins
84	40	β-Selinene	$C_{15}H_{24}$	-	-	-	-	30.36	17066-67-0	Olefins
85	40.1	Cadina-3,5-diene	$C_{15}H_{24}$	-	0.42	-	-	-	267665-20-3	Olefins
86	40.39	Unknown	$C_{15}H_{26}O$	-	-	-	5.05	-		
87	40.43	Cubebol	$C_{15}H_{26}O$	-	0.76	-	-	-	23445-02-5	Alcohols
88	40.76	β-Curcumene	$C_{15}H_{24}$	-	-	-	1.02	-	28976-67-2	Olefins
89	40.77	(-)-Zingiberene	$C_{15}H_{24}$	0.25	0.71	1.13	-	-	495-60-3	Olefins
90	40.81	(Z)-β-Curcumen-12-ol	$C_{15}H_{24}O$	-	-	-	-	0.44	698365-10-5	Alcohols
91	40.99	α-Bergamotene	$C_{15}H_{24}$	-	1.18	-	-	-	17699-05-7	Olefins
92	41	(Z, E)-α-Farnesene	$C_{15}H_{24}$	-	-	-	0.68	-	26560-14-5	Olefins
93	41.12	Unknown	$C_{15}H_{24}$	-	-	-	2.26	-		
94	41.81	β-Bisabolene	$C_{15}H_{24}$	-	-	0.43	0.3	-	495-61-4	Olefins
95	41.87	Widdrol	$C_{15}H_{26}O$	-	0.52	-	-	-	6892-80-4	Alcohols
96	41.95	Unknown	$C_{15}H_{26}O$	1.09	0.92	-	-	-		
97	42.06	7-epi-α-selinene	$C_{15}H_{24}$	0.45	-	-	-	-	123123-37-5	Olefins
98	42.07	Epicubebol	$C_{15}H_{26}O$	-	0.62	-	1.21	-	38230-60-3	Alcohols
99	42.37	Isobornyl 3-methylbutanoate	$C_{15}H_{26}O_2$	-	-	-	-	0.22	7779-73-9	Esters
100	42.46	Drimenol	$C_{15}H_{26}O$	-	0.46	-	-	-	468-68-8	Alcohols
101	42.9	(-)-β-Cadinene	$C_{15}H_{24}$	0.4	2.25	0.25	0.4	0.1	523-47-7	Olefins
102	43.08	β-Sesquiphellandrene	$C_{15}H_{24}$	4.23	5	4.13	3.66	2.07	20307-83-9	Olefins

103	43.54	Cedr-8-ene	$C_{15}H_{24}$	-	0.23	-	-	-	469-61-4	Olefins
104	44.13	Unknown	$C_{15}H_{22}O_2$	0.22	-	-	-	-		
105	44.35	Naphthalene,2,6-dimethyl-	$C_{12}H_{12}$	-	0.47	-	-	-	581-42-0	Aromatic compounds
106	46.34	Bornyl tiglate	$C_{15}H_{24}O_2$	-	-	0.28	-	1.07	101223-92-1	Esters
107	46.36	6-epi-shyobunol	$C_{15}H_{26}O$	-	0.16	-	-	-	69350-61-4	Alcohols
108	46.89	(±)-trans-Nerolidol	$C_{15}H_{26}O$	0.34	-	-	-	-	7212-44-4	Alcohols
109	47.32	Germacrene D-4-ol	$C_{15}H_{26}O$	-	0.42	-	-	-	198991-79-6	Alcohols
110	47.43	Spathulenol	$C_{15}H_{24}O$	0.67	0.34	0.85	1.19	0.19	6750-60-3	Alcohols
111	47.79	Caryophyllene oxide	$C_{15}H_{24}O$	2.45	2.27	1.51	1.7	1.08	1139-30-6	Olefins
112	48.13	Dihydrocurcumene	$C_{15}H_{24}$	-	-	-	-	0.47	1461-02-5	Olefins
113	48.14	aR-Turmerol	$C_{15}H_{22}O$	-	-	0.45	-	-	38142-57-3	Alcohols
114	48.15	Isospathulenol	$C_{15}H_{24}O$	0.39	-	-	0.61	-	88395-46-4	Alcohols
115	48.58	Viridiflorol	$C_{15}H_{26}O$	-	0.4	-	-	0.12	552-02-3	Alcohols
116	48.8	Salvial-4(14)-en-1-one	$C_{15}H_{24}O$	-	-	-	0.25	-	73809-82-2	Ketones
117	48.81	Tricyclo [4.4.0.0(2,7)] dec-3-ene-3-methanol,1-methyl-8-(1-methylethyl)-	$C_{15}H_{24}O$	-	0.34	-	-	-	115728-41-1	Alcohols
118	48.82	cis-Z-α-Bisabolene epoxide	$C_{15}H_{24}O$	0.27	-	-	-	-	121467-35-4	Olefins
119	49.11	Isoshyobunone	$C_{15}H_{24}O$	-	-	-	0.14	-	21698-46-4	Ketones
120	49.17	Cedrane,8-propoxy-	$C_{18}H_{32}O$	-	-	-	0.22	-	19870-75-8	Ether
121	49.61	β-Ionone	$C_{13}H_{20}O$	-	-	0.5	-	-	79-77-6	Ketones
122	50.07	Calarene epoxide	$C_{15}H_{24}O$	-	-	-	0.48	-	68926-75-0	Ether
123	50.09	Humulene epoxide II	$C_{15}H_{24}O$	0.22	-	-	-	0.13	19888-34-7	Olefins
124	50.23	Unknown	$C_{14}H_{22}O$	-	-	-	-	0.28		
125	50.57	Isoaromadendrene epoxide	$C_{15}H_{24}O$	-	-	-	0.45	4.4	499134-59-7	Olefins
126	50.58	Unknown	$C_{15}H_{24}O$	-	0.16	-	-	-		
127	50.69	Junenol	$C_{15}H_{26}O$	-	-	0.82	-	-	472-07-1	Alcohols
128	50.72	(1R,7S, E)-7-Isopropyl-4,10-dimethylenecyclodec-5-enol	$C_{15}H_{24}O$	0.22	0.22	0.17	-	-	81968-62-9	Alcohols

129	51	7-epi-cis-sesquisabinene hydrate	$C_{15}H_{26}O$	-	0.21	0.16	-	-	222638-20-2	Olefins
130	51.58	β-Guaiene	$C_{15}H_{24}$	-	0.19	-	-	-	88-84-6	Olefins
131	51.59	Unknown	$C_{15}H_{22}O$	-	-	-	0.17	-		
132	51.79	Di-epi-1,10-cubenol	$C_{15}H_{26}O$	-	0.74	-	-	-	73365-77-2	Alcohols
133	51.82	Epicubenol	$C_{15}H_{26}O$	0.5	-	-	-	-	19912-67-5	Alcohols
134	52.3	Caryophylla-4(12),8(13)-dien-5α-ol	$C_{15}H_{24}O$	-	0.28	0.17	-	-	19431-79-9	Alcohols
135	52.35	10,10-Dimethyl-2,6-dimethylenebicyclo [7.2.0] undecan-5β-ol	$C_{15}H_{24}O$	0.46	-	-	0.4	-	19431-80-2	Alcohols
136	52.64	(-)-Spathulenol	$C_{15}H_{24}O$	-	0.2	-	-	-	77171-55-2	Alcohols
137	52.86	tau. - Muurolol	$C_{15}H_{26}O$	-	0.76	-	-	-	19912-62-0	Alcohols
138	52.91	tau. - Cadinol	$C_{15}H_{26}O$	0.22	-	-	-	-	5937-11-1	Alcohols
139	52.99	α-acorenol	$C_{15}H_{26}O$	-	-	-	0.74	-	28296-85-7	Alcohols
140	53.17	Unknown	$C_{20}H_{32}$	1.05	-	-	-	-		
141	53.34	Widdrenal	$C_{15}H_{22}O$	-	-	-	0.51	0.36	470-41-7	Aldehydes
142	53.42	Unknown	$C_{15}H_{26}$	-	-	-	1.43	-		
143	53.59	Neointermedeol	$C_{15}H_{26}O$	-	-	-	0.16	-	5945-72-2	Alcohols
144	53.87	Intermedeol	$C_{15}H_{26}O$	9.19	1.36	1.9	4.67	8.45	6168-59-8	Alcohols
145	54.31	Cedren-13-ol,8-	$C_{15}H_{24}O$	-	-	0.48	-	0.19	18319-35-2	Alcohols
146	54.48	Unknown	$C_{15}H_{24}O$	-	-	-	0.99	0.11		
147	55.13	Alloaromadendrene oxide-(2)	$C_{15}H_{24}O$	-	-	-	1	-	25246-27-9	Olefins
148	55.15	cis-Sesquisabinene hydrate	$C_{15}H_{26}O$	-	-	-	-	0.22	58319-05-4	Alcohols
149	55.22	Mustakone	$C_{15}H_{22}O$	-	0.37	-	-	-	1209-91-2	Ketones
150	55.36	Unknown	$C_{17}H_{28}O_3$	0.3	-	-	-	-		
151	55.52	Unknown	$C_{15}H_{22}O$	-	-	0.32	-	-		
152	55.97	α-Bisabolol	$C_{15}H_{26}O$	0.45	4.25	0.27	0.69	0.05	515-69-5	Alcohols
153	56.21	4-(1,5-Dimethylhex-4-enyl) cyclohex-2-enone	$C_{14}H_{22}O$	0.41	0.32	0.36	-	-	1723-80-4	Ketones
154	56.26	Unknown	$C_{20}H_{32}$	-	-	-	-	0.34		
155	56.5	Thunbergol	$C_{20}H_{34}O$	-	-	0.49	-	0.3	25269-17-4	Alcohols

156	56.86	15-Hydroxy-α-muurolene	$C_{15}H_{24}O$	-	0.17	-	-	-	135118-51-3	Olefins
157	56.88	Aromadendrene oxide-(2)	$C_{15}H_{24}O$	-	-	-	1.15	-	85710-39-0	Olefins
158	57.63	6-Isopropenyl-4,8a-dimethyl-1,2,3,5,6,7,8,8a-octahydro-naphthalen-2-ol	$C_{15}H_{24}O$	-	-	2.07	1.18	-	726134-57-2	Alcohols
159	57.84	Aristolene epoxide	$C_{15}H_{24}O$	-	-	-	0.89	-	6831-16-9	Olefins
160	58.08	Unknown	$C_{14}H_{16}$	0.46	-	-	-	-		
161	58.1	Chamazulene	$C_{14}H_{16}$	-	3.5	-	-	-	529-05-5	Terpenoids
162	58.41	Germacron	$C_{15}H_{22}O$	-	-	1.38	-	-	6902-91-6	Ketones
163	59.19	7-Isopropenyl-1,4a-dimethyl-4,4a,5,6,7,8-hexahydro-3H-naphthalen-2-one	$C_{15}H_{22}O$	-	-	-	0.81	0.05	473-08-5	Ketones
164	59.32	(E)-Atlantone	$C_{15}H_{22}O$	-	-	-	-	0.2	108645-54-1	Ketones
165	59.41	2-[4-methyl-6-(2,6,6-trimethylcyclohex-1-enyl) hexa-1,3,5-trienyl] cyclohex-1-en-1-carboxaldehyde	$C_{23}H_{32}O$	-	0.22	-	0.17	-	1033420-57-3	Aldehydes
166	59.78	Alloaromadendrene oxide-(1)	$C_{15}H_{24}O$	-	-	-	0.71	-	85760-81-2	Olefins
167	59.94	Costol	$C_{15}H_{24}O$	0.23	-	0.72	-	0.54	515-20-8	Alcohols
168	60.1	1,1'-biphenyl,2,6-dimethyl-	$C_{14}H_{14}$	-	0.19	-	-	-	3976-34-9	Aromatic compounds
169	60.67	Unknown	$C_{15}H_{22}O_2$	-	-	0.22	-	-		
170	61.33	Methyl 2-((2R,4aR)-4a,8-dimethyl-1,2,3,4,4a,5,6,7-octahydronaphthalen-2-yl) acrylate	$C_{16}H_{24}O_2$	-	-	0.67	-	-	132342-55-3	Esters
171	63.04	Hexahydrofarnesyl acetone	$C_{18}H_{36}O$	0.18	0.45	0.53	1.95	0.15	68607-88-5	Ketones
172	63.18	Unknown	$C_{22}H_{32}$	-	-	-	0.18	-		
173	63.92	(E)-Tonghaosu	$C_{13}H_{12}O_2$	-	-	0.74	-	-	50257-98-2	Ether
174	64.16	Unknown	$C_{21}H_{30}O_2$	-	-	-	-	0.21		
175	65.07	Furan,2,3,5-trimethyl-	$C_7H_{10}O$	0.27	0.48	1.28	0.28	0.11	10504-04-8	Ether
176	65.83	(E)-Tibetin spiroether	$C_{14}H_{14}O_2$	-	-	-	2.45	-	206062-17-1	Ether
177	66.32	n-Hexadecanoic acid	$C_{16}H_{32}O_2$	1.05	0.34	1.12	0.19	0.11	57-10-3	Carboxylic acids

178	67.79	Unknown	$C_{29}H_{42}O$	-	-	0.22	-	-		
179	67.8	Unknown	$C_{15}H_{22}O_2$	-	-	-	-	0.1	352457-43-3	
180	68.83	Isobornyl caprate	$C_{20}H_{36}O_2$	-	-	-	-	0.14	3398-75-2	Esters
181	69.24	Octadecane,3-ethyl-5-(2-ethylbutyl)-	$C_{26}H_{54}$	-	-	-	0.27	-	55282-12-7	Aliphatic Compounds
182	69.93	Linoleic acid	$C_{18}H_{32}O_2$	-	-	0.16	0.26	-	60-33-3	Carboxylic acids
183	70.07	9,12,15-Octadecatrienoic acid, (Z, Z, Z)-	$C_{18}H_{30}O_2$	-	-	0.24	-	-	463-40-1	Carboxylic acids
184	72.98	Eicosane	$C_{20}H_{42}$	0.1	0.06	0.11	0.74	0.14	112-95-8	Aliphatic Compounds
185	75.05	Phenol, 2,2'-methylenebis[6-(1,1-dimethylethyl)-4-methyl-	$C_{23}H_{32}O_2$	-	-	0.12	0.17	-	119-47-1	Phenolic
186	76.29	Tetracosane	$C_{24}H_{50}$	0.95	0.95	0.68	3.03	0.22	646-31-1	Aliphatic Compounds

Table-S5. HPLC fingerprints similarity information of the low molecule fractions compounds.

No.	Values	No.	Values	No.	Values	No.	Values	No.	Values
S1	0.955	S11	0.933	S21	0.884	S31	0.983	S41	0.864
S2	0.956	S12	0.936	S22	0.909	S32	0.983	S42	0.854
S3	0.947	S13	0.934	S23	0.907	S33	0.951	S43	0.886
S4	0.945	S14	0.935	S24	0.958	S34	0.982	S44	0.86
S5	0.947	S15	0.88	S25	0.955	S35	0.984	S45	0.862
S6	0.928	S16	0.938	S26	0.916	S36	0.971	S46	0.864
S7	0.941	S17	0.936	S27	0.928	S37	0.988	S47	0.877
S8	0.962	S18	0.914	S28	0.872	S38	0.985	S48	0.86
S9	0.965	S19	0.937	S29	0.939	S39	0.985	S49	0.864
S10	0.957	S20	0.914	S30	0.924	S40	0.986	S50	0.863

Table-S6. GC fingerprints similarity information of the volatile fractions compounds.

No.	Values	No.	Values	No.	Values	No.	Values	No.	Values
S1	0.519	S11	0.665	S21	0.492	S31	0.682	S41	0.911
S2	0.518	S12	0.678	S22	0.429	S32	0.664	S42	0.911
S3	0.409	S13	0.695	S23	0.422	S33	0.686	S43	0.928
S4	0.411	S14	0.628	S24	0.542	S34	0.692	S44	0.921
S5	0.476	S15	0.663	S25	0.536	S35	0.684	S45	0.92
S6	0.441	S16	0.639	S26	0.462	S36	0.672	S46	0.918
S7	0.514	S17	0.661	S27	0.464	S37	0.689	S47	0.89
S8	0.447	S18	0.641	S28	0.408	S38	0.678	S48	0.917
S9	0.43	S19	0.651	S29	0.382	S39	0.684	S49	0.925
S10	0.537	S20	0.656	S30	0.449	S40	0.677	S50	0.92

Table-S7. GRD of E-tongue and common peaks of the low molecule fractions compounds.

AHS		ANS		CPS		CTS		NMS		PKS		SCS	
**Peak**	**Grade**	**Peak**	**Grade**	**Peak**	**Grade**	**Peak**	**Grade**	**Peak**	**Grade**	**Peak**	**Grade**	**Peak**	**Grade**
**1**	0.869	**1**	0.871	**1**	0.870	**1**	0.883	**1**	0.876	**1**	0.882	**14**	0.817
**4**	0.838	**4**	0.870	**4**	0.863	**4**	0.864	**4**	0.846	**4**	0.882	**13**	0.807
**16**	0.824	**16**	0.839	**16**	0.836	**16**	0.846	**2**	0.828	**16**	0.844	16	0.794
**2**	0.820	**18**	0.825	**18**	0.817	**5**	0.829	**16**	0.828	**18**	0.833	18	0.794
**5**	0.812	**5**	0.816	**2**	0.816	**18**	0.824	**5**	0.807	**5**	0.823	28	0.789
**18**	0.800	**2**	0.810	**5**	0.811	**2**	0.804	**18**	0.803	2	0.795	23	0.781
14	0.786	14	0.780	14	0.775	14	0.795	14	0.778	3	0.785	3	0.772
3	0.774	25	0.778	25	0.775	3	0.789	3	0.767	14	0.782	4	0.770
25	0.769	3	0.777	3	0.771	25	0.77	25	0.765	25	0.775	5	0.770
13	0.754	23	0.764	23	0.757	23	0.765	23	0.746	23	0.772	25	0.767
28	0.747	13	0.745	28	0.740	13	0.756	13	0.743	13	0.749	17	0.766
23	0.745	28	0.744	13	0.740	17	0.745	28	0.742	17	0.744	24	0.761
33	0.735	17	0.735	31	0.733	28	0.744	33	0.735	28	0.737	19	0.753
24	0.735	31	0.732	17	0.730	33	0.737	31	0.731	9	0.733	9	0.746
31	0.725	24	0.732	24	0.730	9	0.735	24	0.731	31	0.728	1	0.712
17	0.725	33	0.729	33	0.728	24	0.734	17	0.725	24	0.727	27	0.709
9	0.716	9	0.722	9	0.718	31	0.722	9	0.714	33	0.727	2	0.699
19	0.712	19	0.711	19	0.708	19	0.716	19	0.712	19	0.708	33	0.686
27	0.689	27	0.688	27	0.686	27	0.695	27	0.686	27	0.688	31	0.640

Table-S8. Regression coefficients of E-tongue and common peaks of the low molecule fractions compounds.

AHS		ANS		CPS		CTS		NMS		PKS		SCS	
**Peak**	**Value**	**Peak**	**Value**	**Peak**	**Value**	**Peak**	**Value**	**Peak**	**Value**	**Peak**	**Value**	**Peak**	**Value**
13	0.1925	4	0.0793	4	0.0723	9	0.4951	19	0.2538	9	0.0999	25	0.2196
25	-0.0961	18	0.0733	13	-0.0712	31	-0.2512	9	0.2335	4	0.0939	14	0.1085
4	-0.0938	23	0.0624	18	0.0627	24	-0.1695	2	0.1449	31	-0.0863	2	-0.0847
19	0.091	17	0.0592	23	0.0493	14	0.1398	13	-0.126	27	-0.0815	9	0.0708
18	-0.0791	27	-0.0537	17	0.0486	23	-0.1373	33	0.1251	18	0.076	19	-0.0705
14	0.0772	19	-0.0534	27	-0.0435	19	0.1209	23	-0.1223	13	-0.0713	18	0.0608
33	-0.0502	9	0.0466	9	0.0353	27	-0.1195	31	-0.1163	33	-0.0648	17	0.0579
9	0.0485	33	-0.0441	24	-0.0351	3	0.1139	14	-0.1141	24	-0.0591	23	0.0497
23	-0.0438	13	-0.044	19	-0.0346	1	0.1113	25	-0.1104	17	0.055	27	0.0221
31	-0.0403	31	-0.0427	31	-0.033	4	-0.086	24	-0.1044	5	0.042	16	0.0172
24	0.0365	5	0.0327	5	0.0268	5	0.0855	16	0.0726	3	0.0377	4	-0.0169
3	0.0308	24	-0.0307	25	0.0239	25	0.0844	1	0.061	23	0.0374	28	0.013
28	0.0285	3	0.0287	3	0.0215	13	-0.0816	5	0.0604	19	-0.0366	3	0.0092
27	0.0273	16	0.0201	16	0.0198	33	-0.0808	3	0.0477	16	0.0347	31	0.0079
16	-0.0207	25	0.0134	14	-0.0197	16	0.0762	4	0.0458	1	0.0219	5	-0.0059
5	0.0177	28	-0.0103	28	-0.0147	2	0.0536	27	-0.0418	28	-0.0193	1	-0.0054
17	-0.011	14	-0.0072	33	-0.0113	28	-0.031	28	-0.0341	25	-0.0187	33	-0.0041
2	-0.006	1	0.0038	2	0.0072	17	0.0274	17	-0.0251	14	-0.0142	24	0.004
1	0.0055	2	-0.0038	1	0.0034	18	-0.0136	18	0.0052	2	0.0083	13	0.0039

Table-S9. Spearman correlation analysis of E-nose and common peaks of the volatile fractions compounds.

Compounds	Odor characteristics	W1C	W5S	W3C	W6S	W5C	W1S	W1W	W2S	W2W	W3S
Peak6	Turpentine	-0.590***	0.800***	-0.680***	-0.013	-0.750***	0.510***	0.600***	0.24	0.730***	-0.25
Peak7	Camphor	-0.570***	0.770***	-0.630***	0.012	-0.700***	0.500***	0.570***	0.25	0.700***	-0.27
Peak19	Flowers fragrance	-0.360**	0.032	-0.27	0.380**	-0.24	0.380**	-0.18	0.390**	-0.021	-0.22
Peak21	Camphor oil	0.098	-0.041	0.088	0.19	0.13	-0.013	0.089	0.016	0.084	0.430**
Peak22	Fruity sweetness	-0.2	0.510***	-0.26	0.082	-0.26	0.26	0.550***	0.13	0.590***	0.27
Peak23	Citrus, lemon	-0.620***	0.780***	-0.640***	0.19	-0.680***	0.590***	0.570***	0.380**	0.740***	-0.15
Peak38	Camphor oil	-0.460***	0.850***	-0.560***	-0.017	-0.620***	0.380**	0.690***	0.15	0.800***	-0.071
Peak40	Camphor oil	-0.690***	0.800***	-0.710***	0.18	-0.740***	0.670***	0.560***	0.450***	0.750***	-0.23
Peak44	Pepper scent, earth scent	-0.640***	0.870***	-0.680***	0.290*	-0.710***	0.640***	0.750***	0.470***	0.890***	0.16
Peak45	Fennel and mint	0.08	0.480***	-0.094	-0.420**	-0.16	-0.21	0.570***	-0.380**	0.480***	-0.023
Peak53	Pine leaves	-0.380**	0.078	-0.330*	0.320*	-0.310*	0.400**	-0.15	0.340*	0.004	-0.23
Peak71	Lila	-0.370**	0.24	-0.280*	0.540***	-0.25	0.480***	0.056	0.490***	0.25	0.18
Peak73	Woody, spicy	-0.27	0.480***	-0.320*	0.11	-0.320*	0.320*	0.530***	0.21	0.570***	0.26
Peak74	Green, floral	-0.550***	0.510***	-0.530***	0.470***	-0.500***	0.600***	0.27	0.520***	0.510***	0.078
Peak83	Piquancy	0.660***	-0.760***	0.700***	0.014	0.740***	-0.610***	-0.570***	-0.400**	-0.700***	0.340*
Peak101	Costus	-0.059	0.380**	-0.19	-0.28	-0.25	-0.059	0.410**	-0.24	0.350*	-0.022
Peak102	--	0.049	0.360**	-0.077	-0.19	-0.15	-0.15	0.380**	-0.290*	0.340*	0.17
Peak110	--	-0.092	0.097	0.012	0.470***	0.023	0.2	0.041	0.26	0.13	0.360**
Peak111	Green grass	-0.1	0.450***	-0.2	-0.17	-0.26	0.02	0.450**	-0.14	0.420**	0.029

Peak144	--	-0.400**	0.670***	-0.440**	0.16	-0.490***	0.380**	0.600***	0.2	0.660***	0.16
Peak152	Faint fragrance	-0.300*	0.340*	-0.27	0.071	-0.290*	0.300*	0.300*	0.21	0.320*	-0.14
Peak171	--	-0.14	0.23	-0.16	-0.14	-0.2	0.065	0.17	-0.027	0.17	-0.16
Peak175	Faint fragrance	-0.21	0.23	-0.28	-0.13	-0.280*	0.16	0.16	0.007	0.19	-0.28
Peak177	Palm	0.2	-0.044	0.15	-0.350*	0.11	-0.290*	-0.015	-0.340*	-0.092	-0.16
Peak184	--	-0.320*	0.290*	-0.300*	0.14	-0.290*	0.360**	0.091	0.280*	0.27	-0.21
Peak186	--	0.370**	-0.23	0.350*	-0.320*	0.340*	-0.460***	-0.16	-0.430**	-0.26	0.033

$^{*}P < 0.05$, $^{**}P < 0.01$, $^{***}P < 0.001$.-- meaning that no reports yet.

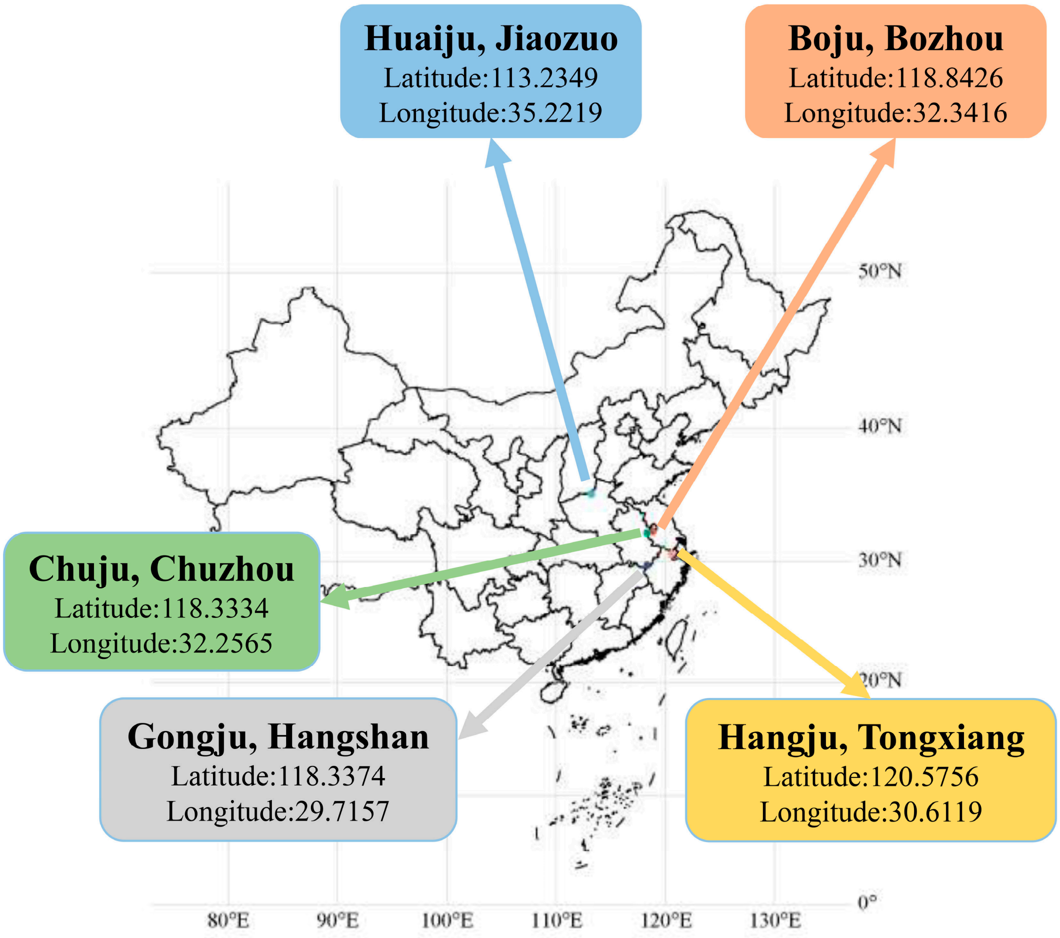

Fig. S1. Traditional geographical origins of the five varieties of Chrysanthemum.

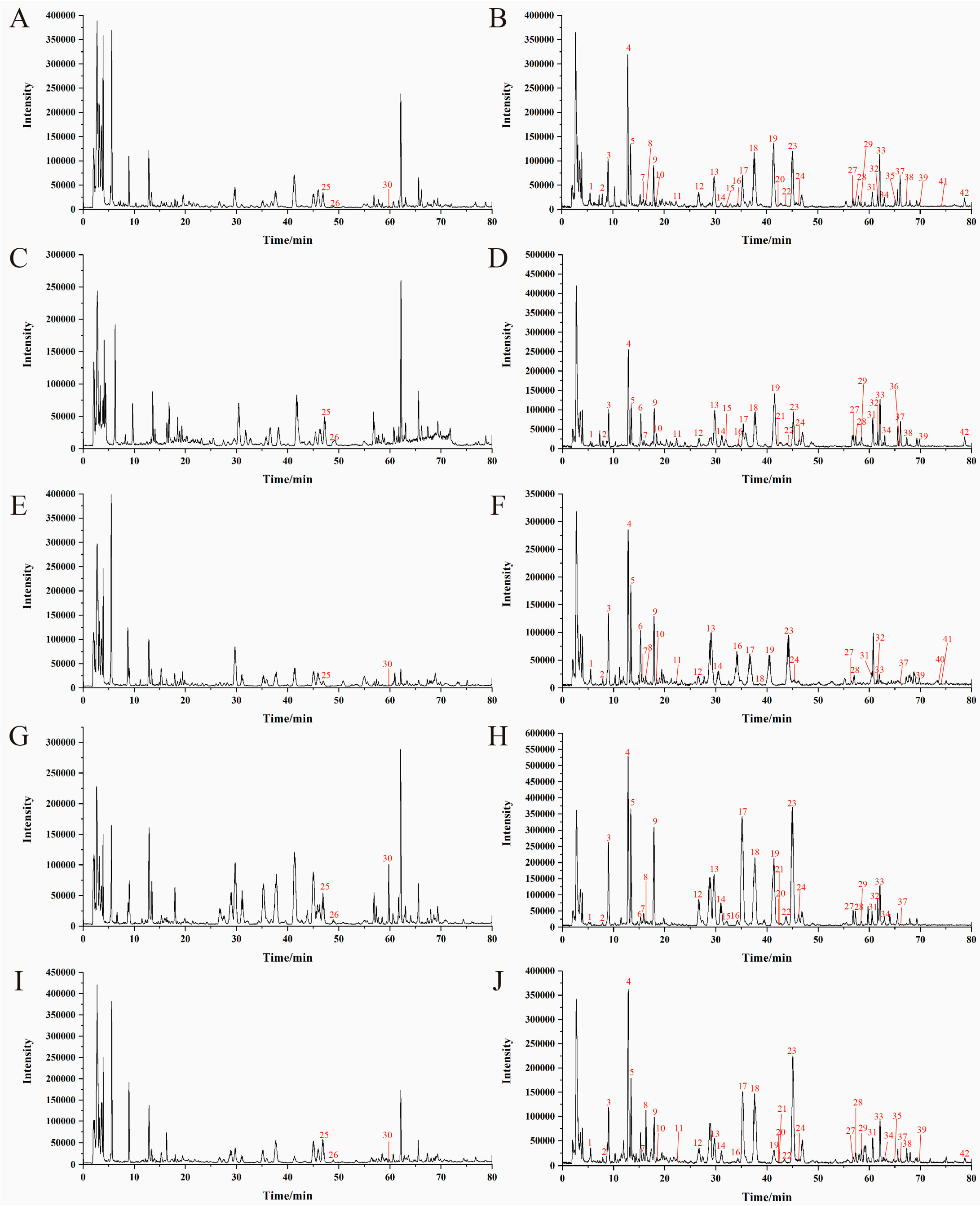

Fig. S2. Total ion chromatograms (TIC) of the low molecule fractions components of five kinds of Chrysanthemums obtained by LC–Q–TOF–MS analysis. (A. Boju-positive ion mode; B. Boju-negative ion mode; C. Chuju-positive ion mode; D. Chuju-negative ion mode; E. Gongju-positive ion mode; F. Gongju-negative ion mode; G. Hangju-Chrysanthemum positive ion mode; H. Hangju-Chrysanthemum negative ion mode; I. Huaijiu-positive ion mode; J. Huaijiu-negative ion mode.).

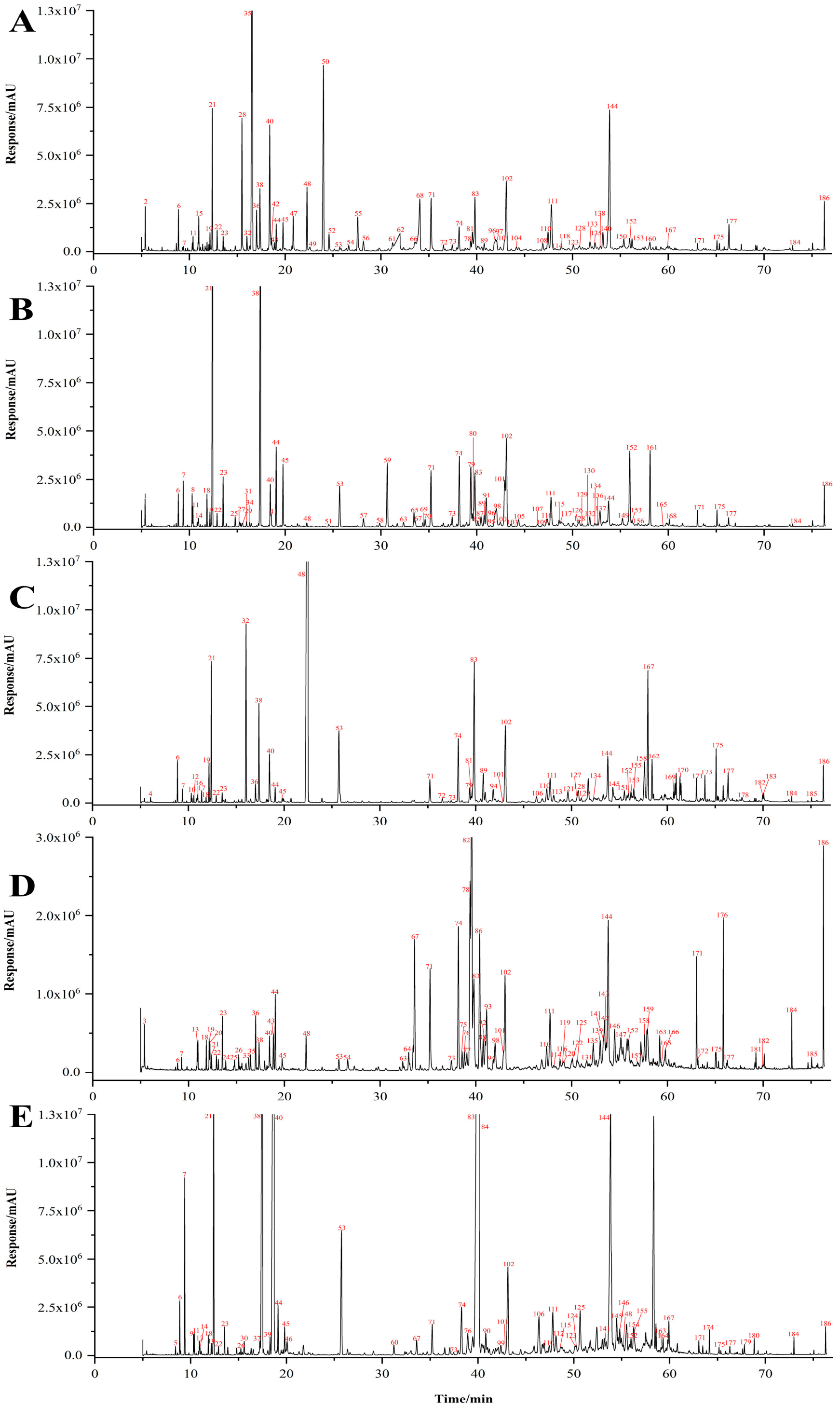

Fig. S3. Identification of the volatile fraction compounds of five kinds of Chrysanthemums obtained by GC–MS analysis. A. Boju; B. Chuju; C. Gongju; D. Hangju; E. Huaijiu.

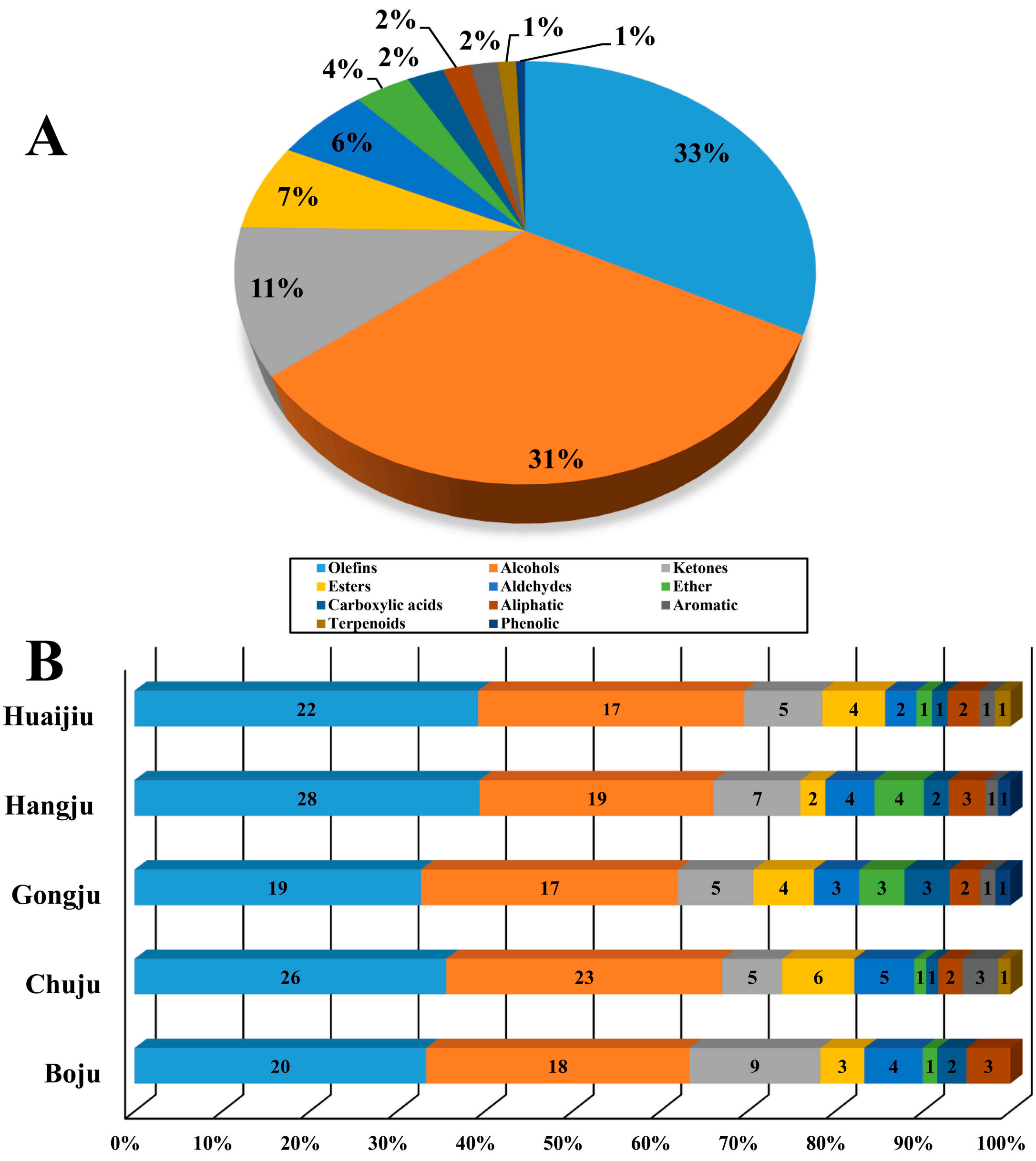

Fig. S4. Types of the volatile fractions compounds for chrysanthemums (A) and five kinds of chrysanthemums (B).

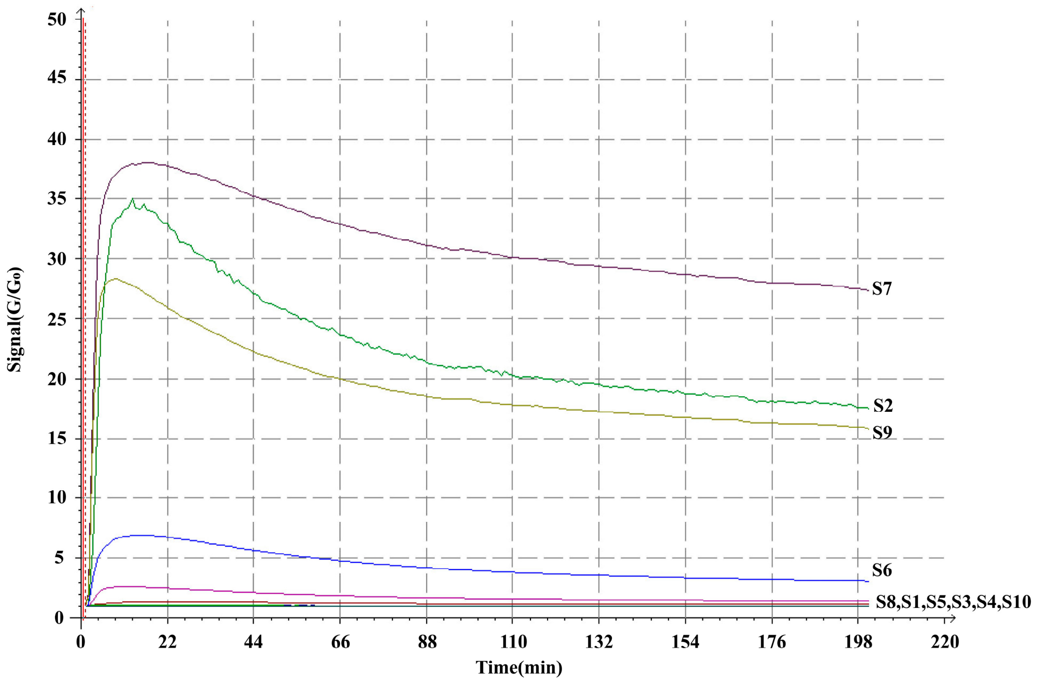

Fig. S5. Response curve of 10 sensors of E-nose.

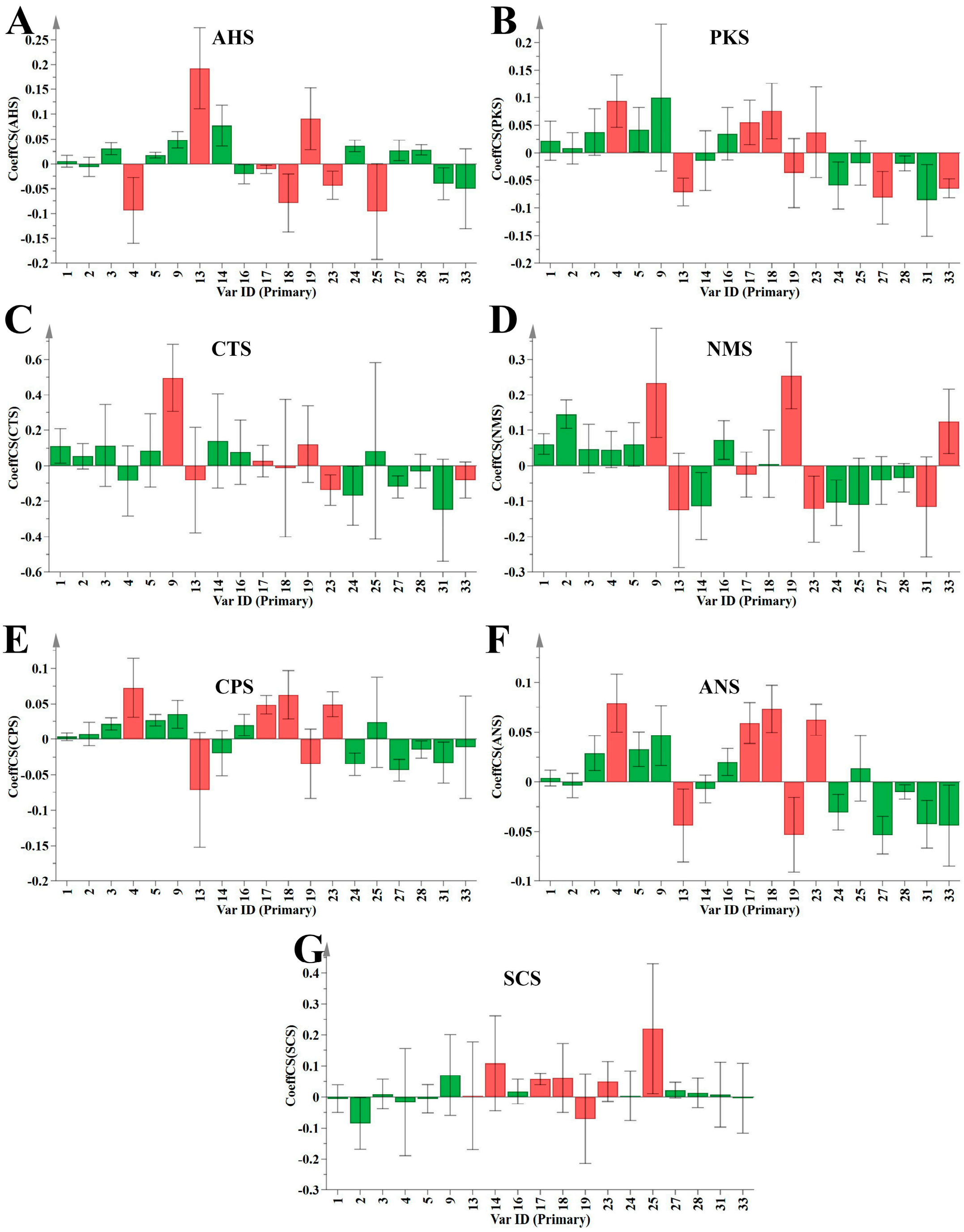

Fig. S6. Regression coefficient plot of seven sensors for E -tongue based on PLSR. (A): AHS; (B): PKS; (C): CTS; (D): NMS; (E): CPS; (F): ANS; (G): SCS.
